# A low-complexity metabolic network model for the respiratory and fermentative metabolism of *Escherichia coli*

**DOI:** 10.1371/journal.pone.0202565

**Published:** 2018-08-29

**Authors:** Ignace L. M. M. Tack, Philippe Nimmegeers, Simen Akkermans, Filip Logist, Jan F. M. Van Impe

**Affiliations:** BioTeC+, Department of Chemical Engineering, KU Leuven, Ghent, Belgium; Maharshi Dayanand University, INDIA

## Abstract

Over the last decades, predictive microbiology has made significant advances in the mathematical description of microbial spoiler and pathogen dynamics in or on food products. Recently, the focus of predictive microbiology has shifted from a (semi-)empirical population-level approach towards mechanistic models including information about the intracellular metabolism in order to increase model accuracy and genericness. However, incorporation of this subpopulation-level information increases model complexity and, consequently, the required run time to simulate microbial cell and population dynamics. In this paper, results of metabolic flux balance analyses (FBA) with a genome-scale model are used to calibrate a low-complexity linear model describing the microbial growth and metabolite secretion rates of *Escherichia coli* as a function of the nutrient and oxygen uptake rate. Hence, the required information about the cellular metabolism (i.e., biomass growth and secretion of cell products) is selected and included in the linear model without incorporating the complete intracellular reaction network. However, the applied FBAs are only representative for microbial dynamics under specific extracellular conditions, viz., a neutral medium without weak acids at a temperature of 37℃. Deviations from these reference conditions lead to metabolic shifts and adjustments of the cellular nutrient uptake or maintenance requirements. This metabolic dependency on extracellular conditions has been taken into account in our low-complex metabolic model. In this way, a novel approach is developed to take the synergistic effects of temperature, pH, and undissociated acids on the cell metabolism into account. Consequently, the developed model is deployable as a tool to describe, predict and control *E. coli* dynamics in and on food products under various combinations of environmental conditions. To emphasize this point,three specific scenarios are elaborated: (*i*) aerobic respiration without production of weak acid extracellular metabolites, (*ii*) anaerobic fermentation with secretion of mixed acid fermentation products into the food environment, and (*iii*) respiro-fermentative metabolic regimes in between the behaviors at aerobic and anaerobic conditions.

## Introduction

In the whole food production and distribution chain, accurate assessment and control of microbiological quality and safety are indispensable. The European Food Safety Authority (EFSA) and the European Centre for Disease Prevention and Control (ECDC) reported 5363 food-borne outbreaks in the EU in 2012, implying the need for more effective control measures [1]. As *E. coli* grows relatively fast on a wide variety of nutrition sources and oxygen concentrations under standard environmental conditions (i.e., a neutral medium with a high water activity at room or body temperature), it is frequently used in experimental microbiological studies. This study focuses on the growth dynamics of *Escherichia coli* K-12 MG1655 on glucose. Abundant information is available about this nonvirulent *E. coli* substrain from systems biology and experimental microbiology, due to its frequent application as a host organism for recombinant DNA [2–7]. In addition, it is a suitable model organism for pathogenic *E. coli* and *Shigella* strains, such as *E. coli* O157:H7 [8] and *S. flexneri* 2a [9]. Infection with these pathogenic strains can result in gastrointestinal disorders, kidney failure and even death. Pathogenic *E. coli* strains are particularly dangerous for young, elderly and immunity-compromised people. An increasing trend of pathogenic *E. coli* infections has been observed in the EU from 2008 to 2012 [1]. In addition, as a facultative anaerobe, *E. coli* can survive both in the presence and absence of oxygen, increasing the risk of food contamination.

To enable adequate food preservation measures, predictive microbiology provides mathematical models to describe and predict microbial dynamics in food products under various environmental conditions resembling food processing and storage [10]. Traditionally, models in predictive microbiology consider the behavior of microbial populations in a (semi-)empirical macroscopic way, and consist of a limited set of coupled differential and algebraic equations [11]. Due to this low-complexity mathematical structure, these population-level models are frequently applied in industry. However, these macroscopic models are only accurate to describe microbial population dynamics where every microorganism is exposed to more or less the same environmental conditions, such as planktonic growth in homogeneous liquids. In contrast, most food products constitute a heterogeneous environment, e.g., semi-solid food structures with nutrient and pH gradients due to colony growth. Hence, in the last decade, the attention of predictive microbiologists has shifted to the behavior of microbial subpopulations and even individual cells, as illustrated by the recent application of the individual-based modeling paradigm in which the microbial cell is taken as the basic modeling unit [12–17]. Mechanistic information about the individual cell metabolism can be obtained from FBAs based on genome-scale models developed in systems biology [18]. However, the incorporation of a genome-scale model in simulations with multiple cells would result in long run times, as these models include a plethora of information about the intracellular metabolic reactions and fluxes. In addition, the metabolic flux distribution is dependent upon the cell objective (e.g., maximization of biomass or metabolite production). This cell objective is often unknown, especially under stressing environmental conditions [19].

In order to map the influence of dynamically changing environmental conditions (e.g., nutrient and oxygen conditions) on the cellular metabolism/growth, FBAs at different values for these dynamic conditions within specific ranges are to be combined to constitue a so-called phenotypic phase plane or PhPP, representing cellular growth or the production of cellular metabolites as a function of the environmental conditions within these ranges [20, 21]. This PhPP is composed as a piecewise plane comprising multiple flat subplanes, each associated with a particular metabolic cellular regime. Therefore, it is possible to develop a low-complexity linear model for the growth and metabolite secretion rates of E. coli cells on glucose and oxygen, based on metabolic FBAs with, e.g., maximization of biomass formulation as the cell objective. More specifically, mechanistic results of the FBAs are used to calibrate a linear model relating the specific cellular growth rate to the specific nutrient uptake rate of the cell. As a result, this linear model contains the intracellular information from the FBAs without explicitly incorporating it. However, these FBAs are only valid when the cell aims to maximize its growth. Deviations from optimal conditions in the culture environment cause shifts in the cell objective and metabolism. For this reason, the synergistic growth-inhibiting effect of high temperatures, low pH values and the presence of undissociated acid cell products in the environment is taken into account in the linear model by means of a novel approach, i.e., the growth-inhibiting effect is not incorporated as a direct adjustment factor to the specific growth rate, but as a set of adjustment factors and terms to the cellular maintenance coefficient and specific nutrient uptake rate. In addition, metabolic shifts as a result of stressing environment conditions are included in the linear model, such as the transition to a lactic acid producing metabolism at low extracellular pH values under anaerobic conditions.

As a result, the developed model is applicable as a low-complexity tool for the description of *E. coli* cells on/in food products under various combinations of environmental factors. This applicability is illustrated by means of three case studies covering all standard metabolic regimes of *E. coli*: (*i*) aerobic respiration as it is the case in well-aerated bioreactors, (*ii*) anaerobic conditions, e.g., in vacuum-packed food products and (*iii*) respiro-fermentative metabolic regimes under micro-aerobic conditions. The performance, advantages, and further applications of the developed modeling approach are summarized in the Discussion section.

## Materials and methods

In this section, two methods to correlate biomass growth and cellular nutrient uptake are compared to each other: (*i*) a noncomplex linear function known as Pirt’s law, and (*ii*) metabolic flux balance analyses based on a genome-based model.

### Traditional approach: Linear correlation between biomass growth and nutrient uptake rate

Microorganisms take up and consume nutrients from their environment, in the first place to fulfill physiological maintenance requirements and, secondly, to support biomass growth and cell reproduction. Maintenance processes comprise osmoregulation, proofreading and internal turnover of macromolecular compounds, cell motility, and defense mechanisms [22]. For the modeling of microbial survival/growth on a single nutrient substrate, these processes are conceptually aggregated as the maintenance coefficient *m*_*S*_ [mol nutrient/(gDW⋅h)] in biochemical models [23]. In addition to this maintenance coefficient, cell growth is determined by the stoichiometric or theoretical biomass yield coefficient *Y*_*X*/*S*_ [gDW/mol nutrient].

The maintenance and biomass yield coefficient link the specific growth rate *μ* [h^−1^] and the specific nutrient uptake rate *q*_*S*_ [mol nutrient/(gDW⋅h)] of the microbial cells in a linear way [23, 24]:

$$\begin{array}{c} \mu =\left(q_{S}-m_{S}\right)\cdot Y_{X/S}. \end{array}$$ (1)

The specific nutrient consumption rate is a function of the extracellular nutrient concentration *C*_*S*_ [mol nutrient/L] according to the Monod kinetics [25]:

$$\begin{array}{c} q_{S}=q_{S,max}\cdot \frac{C_{S}}{K_{S}+C_{S}}, \end{array}$$ (2)

with *q*_*S*,*max*_ the maximum specific nutrient uptake rate [mol nutrient/(gDW⋅h)], and *K*_*S*_ the half-saturation Monod constant for nutrient uptake [mol nutrient/L]. Generally, the half-saturation constant has a very low value (in the order of 10^−6^ M). Hence, it only plays an important role at very low nutrient concentrations.

Analogously to the linear growth law in Eq 1, the secretion of metabolic products into the environment can be expressed as a linear function of the specific nutrient uptake rate:

$$\begin{array}{c} q_{P}=Y_{P/S}\cdot q_{S}+q_{P}{|}_{q_{S}=0}, \end{array}$$ (3)

where *q*_*P*_ [mol product/(gDW⋅h)] is the specific secretion rate of metabolic product *P*, *q*_*P*_ the specific secretion rate at zero cellular nutrient uptake, and *Y*_*P*/*S*_ [mol product/mol nutrient] the yield coefficient of product *P* on nutrient substrate *S*.

### The systems biology approach: Phenotypic phase plane analysis

#### Flux balance analysis

**Fig 1:**
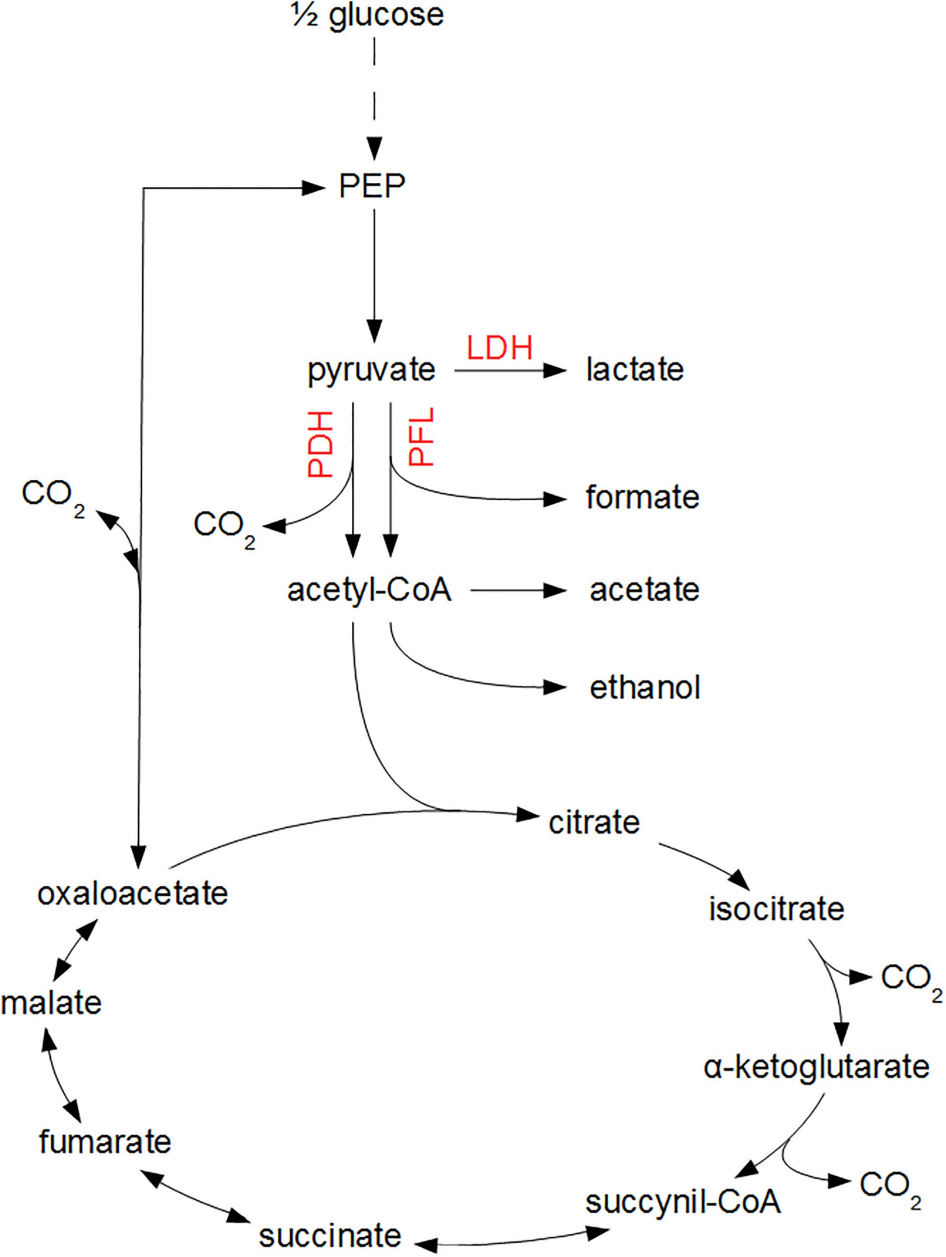
Central metabolic pathways of *E. coli*. Glucose is converted to phosphoenolpyruvate (PEP) and pyruvate through the glycolysis pathway, indicated by the dashed line. Subsequently, under oxygen-rich conditions, pyruvate is converted to acetyl-CoA through the pyruvate dehydrogenase (PDH) enzyme complex, whereupon acetyl-CoA enters the tricarboxylic acid cycle (TCA) as citrate. Under oxygen-limited conditions, pyruvate reacts to lactic acid through the lactate dehydrogenase (LDH) pathway or to formic acid and acetyl-CoA by means of the pyruvate formate lyase (PFL) complex. In the absence of a functional TCA cycle at oxygen limitations, acetyl-CoA is transformed to acetic acid or ethanol.

The mathematical expressions in Eqs (1)–(3) only consider the input-output behavior of individual microorganisms or cell populations, i.e., the dependency of biomass growth and metabolite secretion on nutrient availability. However, these input-output dynamics are the result of complex intracellular reaction networks, as depicted in Fig 1 for the growth of *E. coli* on glucose. Information about the intracellular metabolism and flux distribution is obtained from metabolic flux balance analyses.

Flux balance analysis is a constrained-based mathematical method, based on systems biology concepts, to analyze metabolic reaction networks for the prediction of cellular phenotypes [18]. In systems biology, the intracellular metabolic network is represented by means of the stoichiometric matrix **S**. This matrix maps the intracellular metabolic fluxes vector (**v**) onto a vector containing the time derivatives of the metabolite concentrations (**x**):

$$\begin{array}{c} \frac{dx}{dt}=Sv-\mu \cdot x. \end{array}$$ (4)

The second term in the right-hand side of this equation represents the dilution of intracellular metabolites due to microbial growth. This term is generally neglected, as the fluxes affecting intracellular metabolite concentrations are normally much larger than these concentrations [26]. In this work, the metabolic flux vector should comprise the secretion fluxes together with the intracellular reactions. Therefore, Eq (4) is extended with the external metabolic fluxes (**b**), according to the concept of the exchange stoichiometric matrix **S**_exch_ [18]:

$$\begin{array}{c} \frac{dx}{dt}=S_{\text{exch}}(\begin{array}{c} v\\ b \end{array}). \end{array}$$ (5)

In a FBA, a steady-state solution of Eq (5) is found by optimizing a specific objective function *J*, e.g., biomass growth maximization [20, 21, 27], maximization of metabolite production [28], or a combination of both [29–31]. Mathematically, this is generally expressed as the following optimization problem [18]:

$$\begin{array}{c} \min_{v,b}[J=w\cdot (\begin{array}{c} v\\ b \end{array})], \end{array}$$ (6)

subject to

$$\begin{array}{c} S_{\text{exch}}(\begin{array}{c} v\\ b \end{array})=0, \end{array}$$ (7)

$$\begin{array}{c} 0\leq v_{i}\leq v_{i,max}\;\text{and} \end{array}$$ (8)

$$\begin{array}{c} b_{i,min}\leq b_{i}\leq b_{i,max}. \end{array}$$ (9)

In Eq (6), the weights **w** define the properties of the intended objective. Eqs (8) and (9) respectively express the maximal kinetic rates of the intracellular reactions (*v*_*i*,*max*_) and the physicochemical constraints on the exchange fluxes due to environmental conditions (*b*_*i*,*min*_ and *b*_*i*,*max*_).

#### Phenotypic phase plane analysis

**Fig 2:**
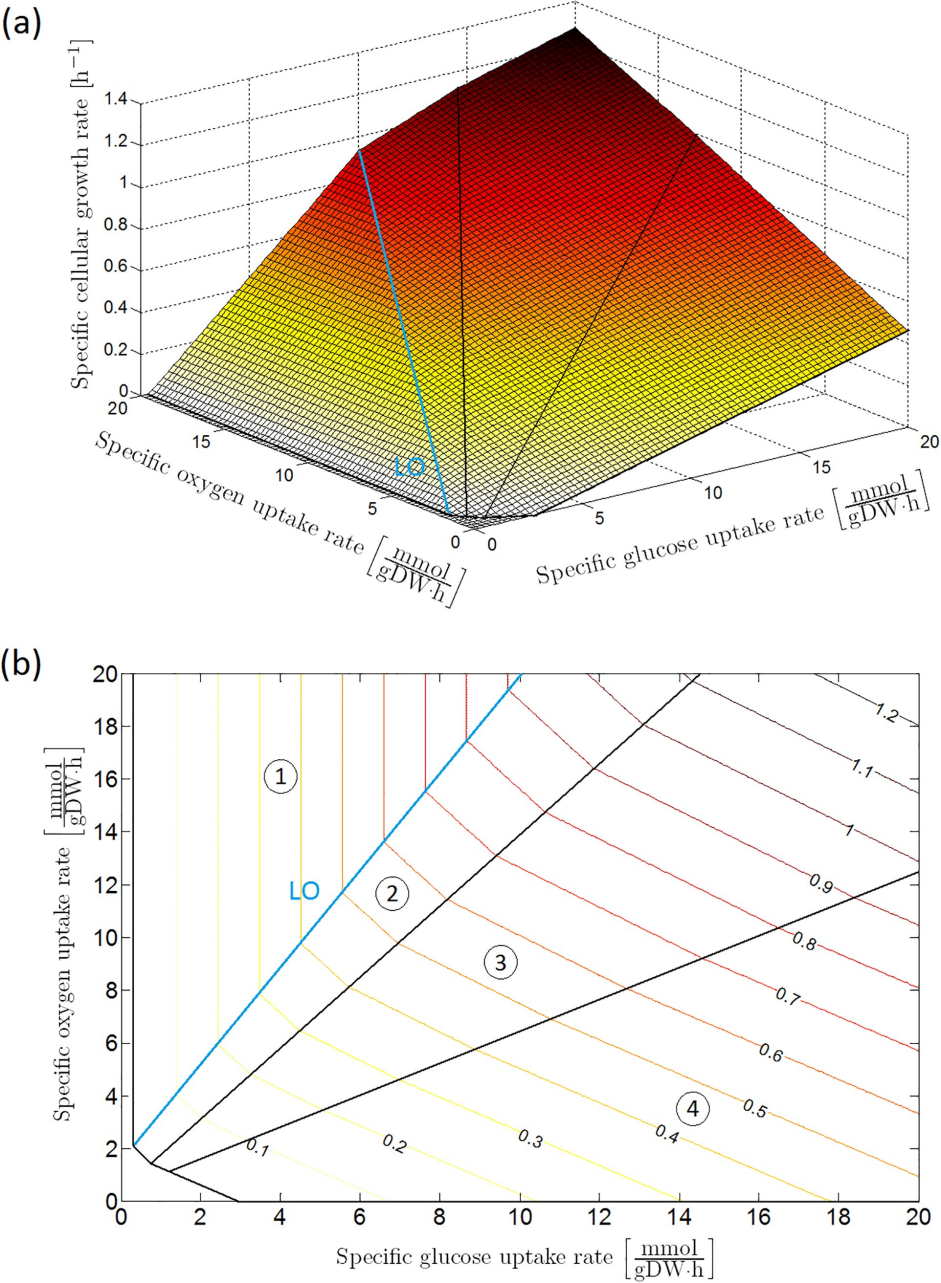
Phenotypic phase plane analysis, after [34]: Specific cellular growth rate as a function of specific glucose and oxygen uptake rates with maximization of biomass growth as cellular objective, presented as (a) 3D plot and (b) contour plot. The phenotypic phase plane consists of four phases, each representing a different metabolic regime. In Sector 1 glucose is completely converted to CO_2_ through the tricarboxylic (TCA) cycle. The other sectors are characterized by the secretion of weak acid cell products in the cellular environment: acetic acid in Sector 2; acetic and formic acid in Sector 3; acetic acid, formic acid and ethanol in Sector 4. On the boundary between Sector 1 and 2, glucose is converted to biomass at a maximal observed yield. For this reason, this boundary is indicated as the line of optimality (LO). This figure has been reprinted from [34]. The original figure has been published under a CC BY license.

Assuming maximization of biomass growth (i.e., minimization of negative growth) as the cellular objective, the specific growth rate on glucose is calculable at each glucose and oxygen uptake rate by means of a FBA using the iAF1260 genome-scale model of [32]. This leads to the phenotypic phase plane (PhPP) of the specific growth rate under reference conditions (i.e., a neutral M9 minimal medium enriched with glucose as carbon source at a temperature of 37 °C) as a function of specific glucose and oxygen consumption rates in Fig 2, determined by means of the COBRA Toolbox in MATLAB^®^ [33].

The obtained phenotypic phase plane consists of four zones: the respiratory metabolism where glycolytic pyruvate is completely converted through the pyruvate dehydrogenase (PDH) enzyme complex towards the tricarboxylic acid cycle (TCA) due to an excess of oxygen as oxidizing agent (Sector 1 in Fig 2(b)), and three respiro-fermentative zones where the oxygen consumption rate is limiting with respect to the oxidation of the assimilated glucose (Sectors 2, 3, and 4). On the boundary line between Sector 1 and 2, the intracellular metabolic network is optimally utilized to maximize the observed biomass yield on glucose (*μ*/*q*_*S*_). Hence, this line is called the *line of optimality* (*LO*). Due to excess glucose uptake with respect to the TCA cycle capacity, acid metabolites are secreted into the environment: acetic acid in Sector 2; acetic and formic acid in Sector 3; acetic acid, formic acid and ethanol in Sector 4.

## Results

The two modeling approaches from the previous section are linked by the phenotypic phase plane concept. In the following section, three case studies will be elaborated based on the obtained phase plane: (*i*) fully aerobic conditions characterized by a respiratory metabolism above the line of optimality (Sector 1 in Fig 2), as it is the case in well-aerated bioreactors, (*ii*) anaerobic conditions, e.g., in vacuum-packed food products and(*iii*) respiro-fermentative metabolic regimes in which weak acid cell products are secreted (Sectors 2, 3, and 4 in Fig 2). A schematic overview of the metabolic models developed for the first two case studies is included in S1 Text.

### Case study I: Respiratory metabolism

#### Respiratory growth on glucose at reference conditions

**Fig 3:**
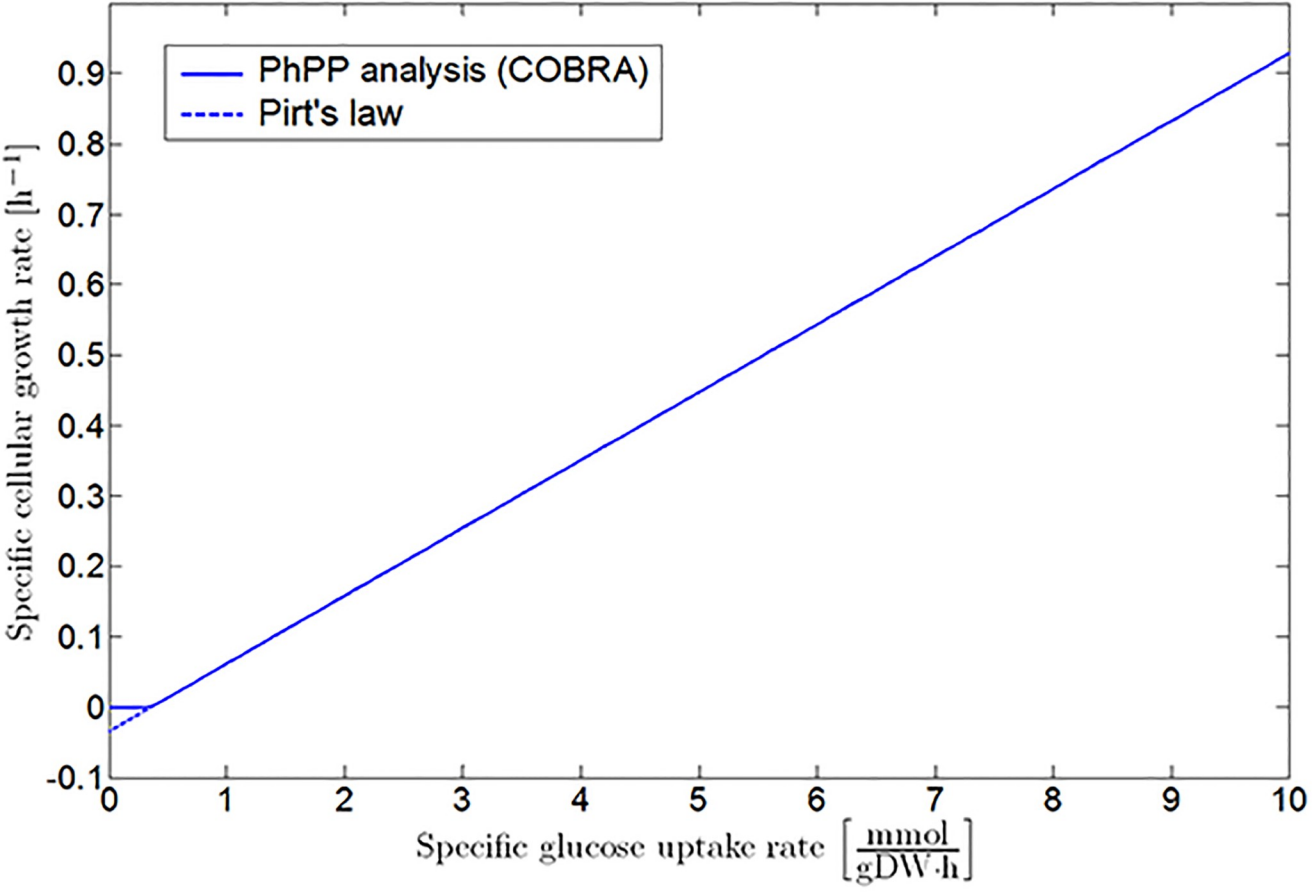
Specific growth rate as a function of specific glucose uptake rate under fully aerobic conditions. The full line represents the results obtained by the PhPP analysis with the COBRA toolbox. These results are adapted to the linear mathematical structure of Pirt’s law, as indicated by the dashed line.

The PhPP in Fig 2 forms the link between the detailed iAF1260 genome-scale model and the classic Pirt’s law [23, 24], as the projection of Sector 1 and the LO on a plane perpendicular to the oxygen uptake axis is described by Eq (1) (see Fig 3). It should be noted that the COBRA PhPP analysis does not take into account the existence of negative growth rates predicted by Pirt’s law. Hence, the COBRA profile is adapted to fit Eq (1), as illustrated with the dotted line in Fig 3. This leads to the following mathematical correlation for *E. coli* K-12 growth on glucose at reference environmental conditions:

$$\begin{array}{c} \mu_{ref}^{\left(1\right)}=(q_{G}-m_{G,ref}^{\left(1\right)})\cdot Y_{X/G}^{\left(1\right)}=(q_{G,max}^{a}\cdot \frac{C_{G}}{K_{G}+C_{G}}-m_{G,ref}^{\left(1\right)})\cdot Y_{X/G}^{\left(1\right)} \end{array}$$ (10)

with $\mu_{ref}^{\left(1\right)}$ [h^−1^] the specific growth rate in Sector 1 of the PhPP at reference conditions, and $q_{G,max}^{a}$ the maximum specific glucose uptake under aerobic conditions. As a mather of fact, this correlation implicitly includes the intracellular information of the iAF1260 model while maintaining the simple mathematical structure of Pirt’s law. Values for the parameters in this equation are listed in Table 1.

**Table 1:** Parameter values in the metabolic models.

Symbol	Explanation	Value	Reference
$E_{a,g}^{a}$	Microbial growth activation energy under aerobic reference conditions^a^	6.1334 ⋅10^4^ J/mol	Derived from data in Fig 4
$E_{a,m}^{a}$	Maintenance activation energy under aerobic reference conditions^a^	3.4664 ⋅10^5^ J/mol	Derived from data in Fig 4
[H^+^]_*min*_	Minimum inhibitory proton concentration under reference conditions^a^	10^−3.95^ mol/L	[35]
*K*_*G*_	Monod half-saturation constant for glucose	2.994 ⋅10^−6^ mol/L	[36]
$m_{G,ref}^{\left(1\right)}$	Maintenance coefficient of the LO under reference conditions^a^	3.496 ⋅10^−4^ $\frac{\text{mol}}{\text{gDW}\cdot h}$	FBA with iAF1260 model
$m_{G,ref}^{LDH}$	Maintenance coefficient for the LDH metabolism under reference conditions^a^	4.195 ⋅10^−3^ $\frac{\text{mol}}{\text{gDW}\cdot h}$	FBA with iAF1260 model
$m_{G,ref}^{PFL}$	Maintenance coefficient for the PFL metabolism under reference conditions^a^	3.051 ⋅10^−3^ $\frac{\text{mol}}{\text{gDW}\cdot h}$	FBA with iAF1260 model
$q_{A,ref}^{PFL}{|}_{q_{G}=0}$	Specific acetic acid secretion rate under glucose-free reference conditions^a^	6.073 ⋅10^−4^ $\frac{\text{mol}}{\text{gDW}\cdot h}$	FBA with iAF1260 model
$q_{F,ref}^{PFL}{|}_{q_{G}=0}$	Specific formic acid secretion rate under glucose-free reference conditions^a^	9.174 ⋅10^−4^ $\frac{\text{mol}}{\text{gDW}\cdot h}$	FBA with iAF1260 model
$q_{G,max}^{a}$	Maximum specific glucose uptake rate under aerobic conditions	9.02 ⋅10^−3^ $\frac{\text{mol}}{\text{gDW}\cdot h}$	[37]
$q_{G,max}^{an}$	Maximum specific glucose uptake rate under anaerobic conditions	17.3 ⋅10^−3^ $\frac{\text{mol}}{\text{gDW}\cdot h}$	[37]
$q_{L,ref}^{LDH}{|}_{q_{G}=0}$	Specific lactic acid secretion rate under glucose-free reference conditions^a^	1.2043 ⋅10^−3^ $\frac{\text{mol}}{\text{gDW}\cdot h}$	FBA with iAF1260 model
${\left[U_{A}\right]}_{min}^{a}$	Mean aerobic MIC of undissociated acetic acid in a pH range from 4.2 to 5.4	9.5 ⋅10^−3^ mol/L	[38]
${\left[U_{F}\right]}_{min}^{a}$	Mean aerobic MIC of undissociated formic acid in a pH range from 4.2 to 5.4	0.95 ⋅10^−3^ mol/L	[38]
${\left[U_{L}\right]}_{min}^{a}$	Mean aerobic MIC of undissociated lactic acid in a pH range from 4.2 to 5.4	7.0 ⋅10^−3^ mol/L	[38]
${\left[U_{A}\right]}_{min}^{an}$	Mean anaerobic MIC of undissociated acetic acid in a pH range from 4.2 to 5.4	6.25 ⋅10^−3^ mol/L	[38]
${\left[U_{F}\right]}_{min}^{an}$	Mean anaerobic MIC of undissociated formic acid in a pH range from 4.2 to 5.4	1.075 ⋅10^−3^ mol/L	[38]
${\left[U_{L}\right]}_{min}^{an}$	Mean anaerobic MIC of undissociated lactic acid in a pH range from 4.2 to 5.4	4.75 ⋅10^−3^ mol/L	[38]
$Y_{X/G}^{\left(1\right)}$	Biomass yield coefficient on glucose under fully aerobic conditions	96.300 gDW/mol	FBA with iAF1260 model
$Y_{L/G}^{LDH}$	Lactic acid yield coefficient on glucose for the LDH metabolism	1.713 mol/mol	FBA with iAF1260 model
$Y_{X/G}^{LDH}$	Biomass yield coefficient on glucose for the LDH metabolism	19.465 gDW/mol	FBA with iAF1260 model
$Y_{A/G}^{PFL}$	Acetic acid yield coefficient on glucose for the PFL metabolism	0.801 mol/mol	FBA with iAF1260 model
$Y_{F/G}^{PFL}$	Formic acid yield coefficient on glucose for the PFL metabolism	1.699 mol/mol	FBA with iAF1260 model
$Y_{X/G}^{PFL}$	Biomass yield coefficient on glucose for the PFL metabolism	27.075 gDW/mol	FBA with iAF1260 model

^a^ Reference conditions: M9 minimal medium enriched with glucose, pH = 7.0, *T* = 37 °C, and [U_*i*_] = 0 mol/L.

#### Influence of extracellular pH and undissociated acid cell products on maintenance requirements

Maintenance requirements are influenced by the environmental pH. Decreasing pH values exhibit an inhibitory effect on microbial growth, proportional to the external proton concentration [39, 40]. Weak organic acids, such as acetic, formic and lactic acid have an additional restrictive influence, mainly in their undissociated form [41]. The lipophilic undissociated acid is able to pass through the cell membrane, disturbing the transmembrane pH gradient and disrupting the concomitant proton-motive force upon intracellular dissociation [42]. It is hypothesized that growth inhibition by weak acids is linearly dependent on the concentration of the undissociated acid form, similarly to the aforementioned proton effect. This leads to the following mathematical expression for the maintenance coefficient:

$$\begin{array}{c} m_{G}^{\left(1\right)}=m_{G,ref}^{\left(1\right)}+A^{\left(1\right)}\cdot \frac{\left[H^{+}\right]-10^{-7}}{{\left[H^{+}\right]}_{\text{min}}-10^{-7}}+\sum_{i}B_{i}^{\left(1\right)}\cdot \frac{\left[U_{i}\right]}{{\left[U_{i}\right]}_{\text{min}}^{a}}. \end{array}$$ (11)

In this equation, the maintenance coefficient is split up in three terms: (*i*) the reference maintenance coefficient $m_{G,ref}^{\left(1\right)}$ at neutral pH and zero weak acid concentrations in the food system, (*ii*) an additional maintenance term due to increasing environmental proton concentrations [H^+^], and (*iii*) supplementary maintenance requirements due to the presence of undissociated lipophilic acids [U_*i*_] in the environment.

To determine the value of the parameters *A*^(1)^ and $B_{i}^{\left(1\right)}$, this expression for the maintenance coefficient is replaced in Eq (1). In a glucose-rich environment (i.e., *q*_*G*_ ≈ *q*_*G*,*max*_) without weak acids, the microbial growth rate decreases to zero when the environmental proton concentration reaches a minimum inhibitory value [H^+^]_*min*_. Hence, the coefficient of the proton maintenance term *A*^(1)^ is calculable from Eq (11):

$$\begin{array}{c} (q_{G,max}^{a}-m_{G,ref}^{\left(1\right)}-A^{\left(1\right)})\cdot Y_{X/G}^{\left(1\right)}=0\Rightarrow A^{\left(1\right)}=q_{G,max}^{a}-m_{G,ref}^{\left(1\right)}. \end{array}$$ (12)

Similarly, ${\left[U_{i}\right]}_{min}^{a}$ is the minimum inhibitory concentration (MIC) of the undissociated form of weak acid *i* in an aerobic nutrient-rich system. Decreases in the intracellular pH due to weak acid dissociation in the cell are associated to significant increases in the cellular glucose uptake rate [43]. It is inferred that this surplus glucose consumption is conducted through the Embden-Mayerhof-Parnas (EMP) glycolytic pathway (see Fig 1), resulting in higher ATP production to fulfill the additional maintenance requirements. From experimental data for *E. coli* K-12 of [43], it is derived that, per molar of undissociated acetic acid in the food system, the specific glucose uptake rate increases at a rate Δ*q*_*G*/*A*_ of 0.025575 $\frac{\text{mol}\;\text{glucose}}{\text{gDW}\cdot h\cdot M\;\text{acetate}}$. Taking this increase in glucose uptake into account, the coefficient $B_{A}^{\left(1\right)}$ of the acetic acid maintenance term can be derived from the mean MIC of undissociated acetic acid under aerobic conditions ${\left[U_{A}\right]}_{min}^{a}$ in a pH range from 4.2 to 5.4, experimentally measured by [38]:

$$\begin{array}{c} (q_{G,max}^{a}+\Delta q_{G/A}\cdot {\left[U_{A}\right]}_{min}^{a}-m_{G,ref}^{\left(1\right)}-\\ A^{\left(1\right)}\cdot \frac{10^{-4.2}+10^{-5.4}-2\cdot 10^{-7}}{2\cdot ({\left[H^{+}\right]}_{min}-10^{-7})}-B_{A}^{\left(1\right)})\cdot Y_{X/G}^{\left(1\right)}=0, \end{array}$$ (13)

resulting in

$$\begin{array}{c} B_{A}^{\left(1\right)}=q_{G,max}^{a}+\Delta q_{G/A}\cdot {\left[U_{A}\right]}_{min}^{a}-m_{G,ref}^{\left(1\right)}-A^{\left(1\right)}\cdot \frac{10^{-4.2}+10^{-5.4}-2\cdot 10^{-7}}{2\cdot ({\left[H^{+}\right]}_{min}-10^{-7})}. \end{array}$$ (14)

For other weak acids, it is hypothetized that the increase in glucose uptake rate at their MIC is equal to the rise in glucose consumption at the aerobic MIC of acetic acid:

$$\begin{array}{c} \Delta q_{G/i}\cdot {\left[U_{i}\right]}_{min}^{a}=\Delta q_{G/A}\cdot {\left[U_{A}\right]}_{min}^{a}. \end{array}$$ (15)

Hence, the $B_{i}^{\left(1\right)}$ coefficients have the same value for each weak acid. Experimental values for the mean MIC in the pH range from 4.2 to 5.4 of formic and lactic acid from [38] are summarized in Table 1.

#### Influence of temperature on the aerobic cellular metabolism

In the normal physiological temperature range (NPTR) for *E. coli* from 21°C to 37°C, the dependence of microbial growth on the environmental temperature can be described by Arrhenius kinetics [44]:

$$\begin{array}{c} \mu^{\left(1\right)}=\mu^{(1),\infty }\cdot \exp(-\frac{E_{a,g}}{R\cdot T}), \end{array}$$ (16)

with *μ*^(1),∞^ the specific growth rate under fully aerobic conditions at infinite temperature, *E*_*a*,*g*_ the microbial growth activation energy $\left[\frac{J}{\text{mol}}\right]$, *R* the universal gas constant $\left[8.3145\frac{J}{\text{mol}\cdot K}\right]$, and *T* the temperature [K].

As both the maintenance and biomass yield coefficient are approximately constant with respect to temperature in the NPTR for *E. coli* [45], the maximum specific glucose consumption rate $q_{G,max}^{a}$ exhibits approximately an Arrhenius-type behavior with respect to temperature as well, according to Pirt’s law (Eq (10)):

$$\begin{array}{c} q_{G,max}^{a}=\frac{\mu^{(1),\infty }}{Y_{X/G}^{\left(1\right)}}\cdot \exp(-\frac{E_{a,g}^{a}}{R\cdot T})+m_{G,ref}^{\left(1\right)}, \end{array}$$ (17)

In addition, the reference maintenance coefficient is relatively small with respect to the maximum specific glucose uptake rate in the NPTR, so that

$$\begin{array}{c} q_{G,max}^{a}\approx q_{G,max}^{a,\infty }\cdot \exp(-\frac{E_{a,g}^{a}}{R\cdot T}), \end{array}$$ (18)

The maximum specific glucose uptake rate at infinite temperature, $q_{G,max}^{a,\infty }$, can be calculated from the maximum specific glucose uptake rate at a reference temperature *T*_*ref*_ of 310.15 K (37 °C):

$$\begin{array}{c} q_{G,max}^{a,\infty }=q_{G,max}^{a}{|}_{T=T_{ref}}\cdot \exp(\frac{E_{a,g}^{a}}{R\cdot T_{ref}}) \end{array}$$ (19)

At temperatures higher than the NPTR, the maintenance coefficient increases rapidly according to the Arrhenius kinetic model, while the biomass yield coefficient is not significantly affected [45, 46]. This leads to a double Arrhenius model for the specific microbial growth rate, according to Pirt’s law:

$$\begin{array}{c} \mu^{\left(1\right)}=(q_{G,max}^{a,\infty }\cdot \exp(-\frac{E_{a,g}^{a}}{R\cdot T})-m_{G,ref}^{\left(1\right)})\cdot Y_{X/G}^{\left(1\right)}\\ \text{for}\;21^{{}^{\circ}}C\leq T<37^{{}^{\circ}}C, \end{array}$$ (20)

$$\begin{array}{c} \mu^{\left(1\right)}=(q_{G,max}^{a,\infty }\cdot \exp(-\frac{E_{a,g}^{a}}{R\cdot T})-m_{G}^{(1),\infty }\cdot \exp(-\frac{E_{a,m}^{a}}{R\cdot T}))\cdot Y_{X/G}^{\left(1\right)}\\ \text{for}T\geq 37^{{}^{\circ}}C, \end{array}$$ (21)

with $m_{G}^{(1),\infty }$ the maintenance coefficient at infinite temperature, and $E_{a,m}^{a}$ the maintenance activation energy under aerobic conditions. The maintenance coefficient $m_{G}^{(1),\infty }$ at infinite temperature is calculated in an analogous way to the maximum specific glucose uptake at infinite temperature (Eq (19)):

$$\begin{array}{c} m_{G}^{(1),\infty }=m_{G,ref}^{\left(1\right)}\cdot \exp(\frac{E_{a,m}^{a}}{R\cdot T_{ref}}). \end{array}$$ (22)

**Fig 4:**
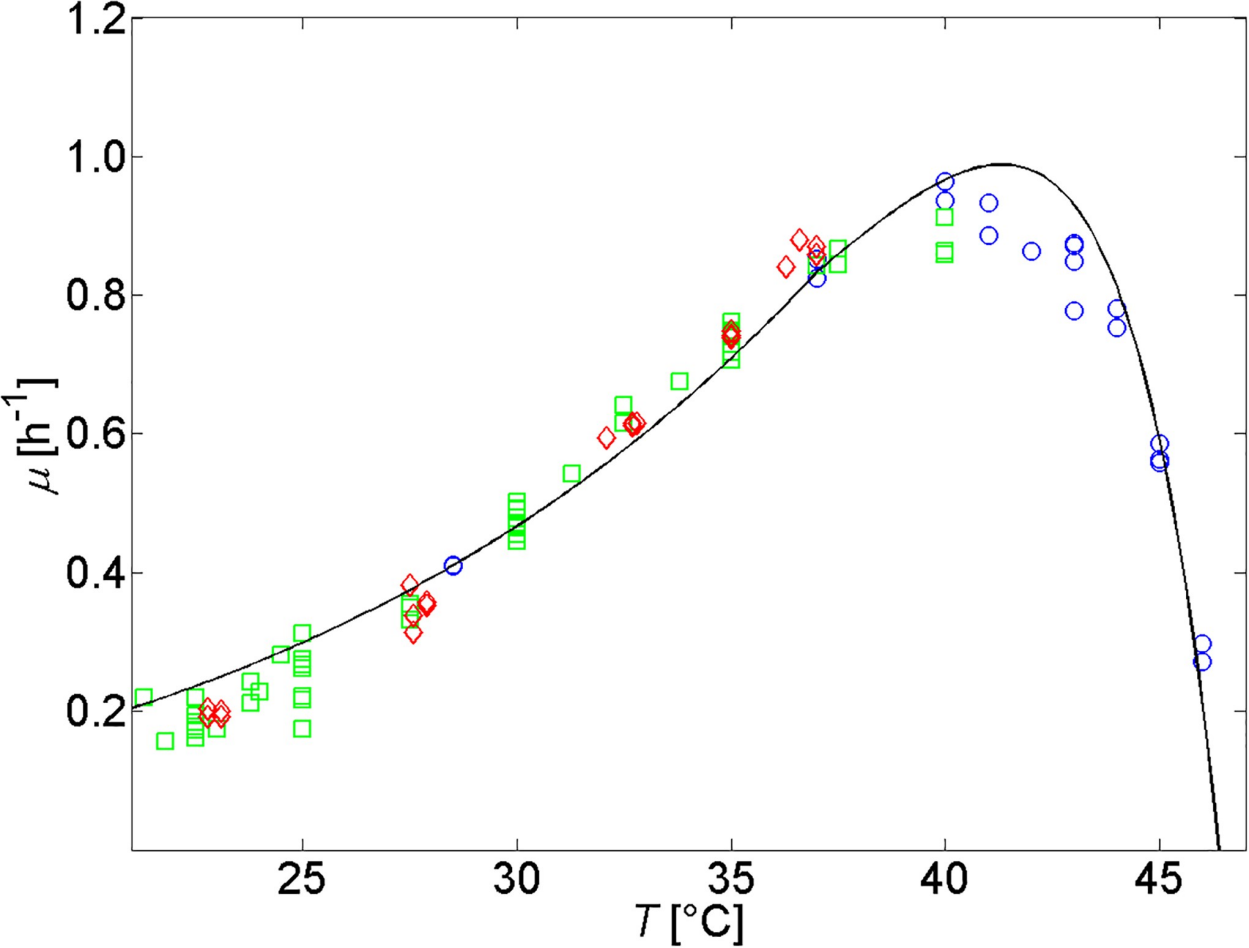
Specific microbial growth rate as a function of temperature according to Eqs (20) and (21). Experimental data are taken from [47] (◊), [48] (▫), and [49] (∘). A rescaling factor of 0.382 is used to take into account that these data were obtained from experiments in BHI medium supporting higher specific growth rates than M9 media [37, 49].

Growth and maintenance activation energies $E_{a,g}^{a}$ and $E_{a,m}^{a}$ are derived from experimental growth data for *E. coli* K-12 MG1655 of [47, 48] and [49], as presented in Fig 4. The obtained value for $E_{a,m}^{a}$ of 346.64 kJ/mol (see Table 1) matches with the range of 212-515 kJ/mol determined by [45].

The developed expression for temperatures above 37 °C (Eq (21)) is particularly similar to the Hinshelwood equation for the influence of temperature on the specific microbial growth rate [50]:

$$\begin{array}{c} \mu =k_{1}\cdot \exp(-\frac{E_{1}}{R\cdot T})-k_{2}\cdot \exp(-\frac{E_{2}}{R\cdot T}). \end{array}$$ (23)

In this model, the microbial growth rate is controlled by a single enzyme reaction. This enzyme reaction produces a heat-sensitive product that is irreversibly denatured at high temperatures, as represented by the second exponential term. The scaling constants *k*_1_ and *k*_2_ [h^−1^], and activation energies *E*_1_ and *E*_2_ [$\frac{J}{\text{mol}}$] define the microbial growth and denaturation reaction, respectively. It should be noted that in this kind of Hinshelwood models the microbial growth does not become negative at low temperatures. Unfortunately, the effect of low temperatures on the parameters in Pirt’s law (Eq 1) are not known from literature.

### Case study II: Anaerobic conditions

#### Anaerobic microbial growth on glucose at reference environmental conditions

**Fig 5:**
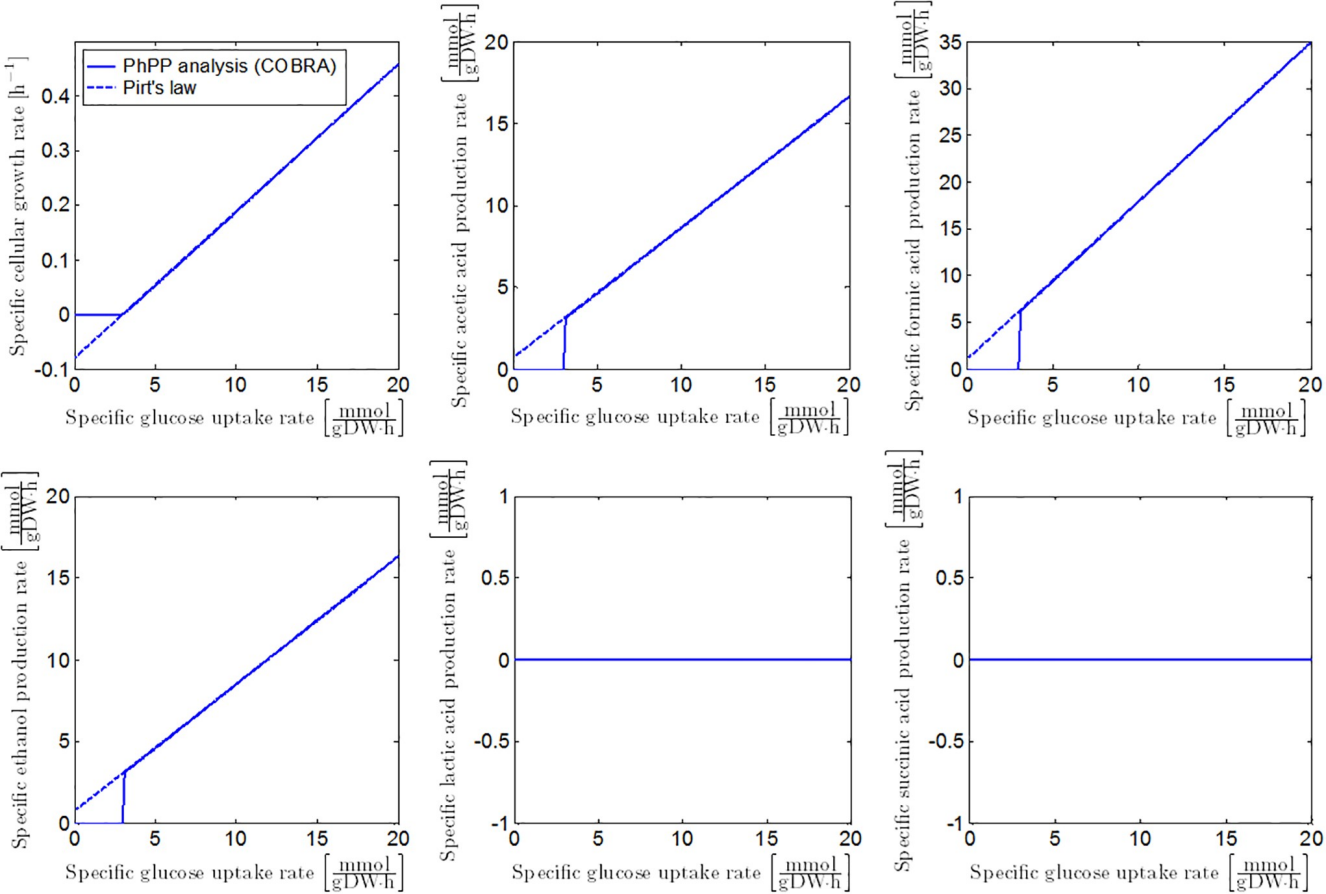
Specific growth rate and metabolite secretion rates as a function of specific glucose uptake rate. Full lines represent the FBA results with the iAF1260 model. Dashed lines illustrate the model of [23] fitted on the FBA output.

**Fig 6:**
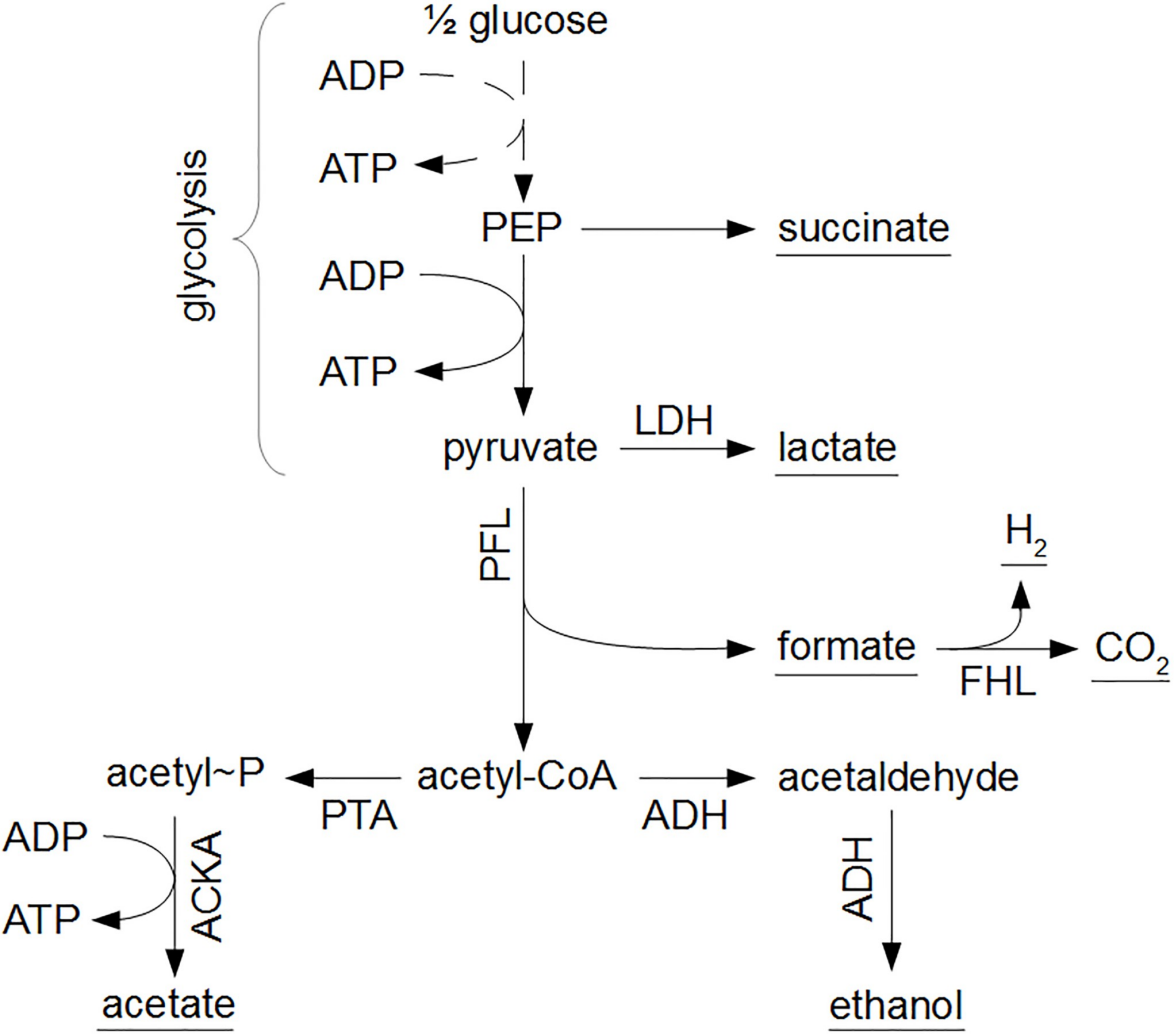
Anaerobic metabolism of *E. coli*. From phosphoenolpyruvate (PEP) and pyruvate, metabolic products are formed in reactions catalyzed by lactate dehydrogenase (LDH), pyruvate formate lyase (PFL), phosphotransacetylase (PTA), acetate kinase (ACKA), alcohol dehydrogenase (ADH), and formate hydrogen lyase (FHL). The underlined metabolic products are secreted to the environment.

Similar to the procedure in Case Study I, anaerobic growth dynamics of *E. coli* can be obtained from fitting the PhPP with Pirt’s law, as illustrated in Fig 5. According to the PhPP analysis, under optimal anaerobic growth conditions, glycolytic phosphoenolpyruvate (PEP) is completely converted to pyruvate and subsequently through the pyruvate formate lyase (PFL) reaction (Fig 6). This results in the secretion profiles of acetic acid, ethanol, and formic acid in Fig 5. The following mathematical correlations describe *E. coli* K-12 growth on glucose at reference anaerobic environmental conditions, when the glycolytic PEP is completely converted through the PFL reaction:

$$\begin{array}{ccc} \mu_{ref}^{PFL} & = & \left(q_{G}-m_{G,ref}^{PFL}\right)\cdot Y_{X/G}^{PFL} \end{array}$$ (24)

$$\begin{array}{ccc} q_{P,ref}^{PFL} & = & Y_{P/G}^{PFL}\cdot q_{G}+q_{P,ref}^{PFL}{|}_{q_{G}=0} \end{array}$$ (25)

with $\mu_{ref}^{PFL}$ [h^−1^] the specific growth rate at reference conditions, and $q_{P,ref}^{PFL}$ [mol/(gDW⋅h)] the reference specific secretion rate of metabolic product *P* (acetic acid, ethanol or formic acid). The specific metabolite secretion rates are linearly related to the specific glucose uptake rate *q*_*G*_ by means of a product yield coefficient $Y_{P/G}^{PFL}$. Values for the parameters in these equations are summarized in Table 1. The positive values for the specific metabolite secretion rates at zero glucose uptake can be explained by presuming that cellular biomass is converted into cell products at this zero glucose uptake in order to satisfy the maintenance requirements of the cell.

#### Influence of the extracellular pH on the conversion of glycolytic pyruvate to metabolic products

Under neutral anaerobic conditions, pyruvate formate lyase (PFL) catalyses the reaction from pyruvate to acetyl-CoA and formic acid, according to the microbial growth maximization objective. One molecule of acetic acid or ethanol is formed from acetyl-CoA. To maintain the cellular NAD^+^/NADH redox balance, approximately equal amounts of acetic acid and ethanol are generated [51, 52]. The results of the PhPP analysis are in accordance to this 2:1:1 ratio between the formic acid, acetic acid and ethanol secretion rates, as illustrated in Fig 5.

**Fig 7:**
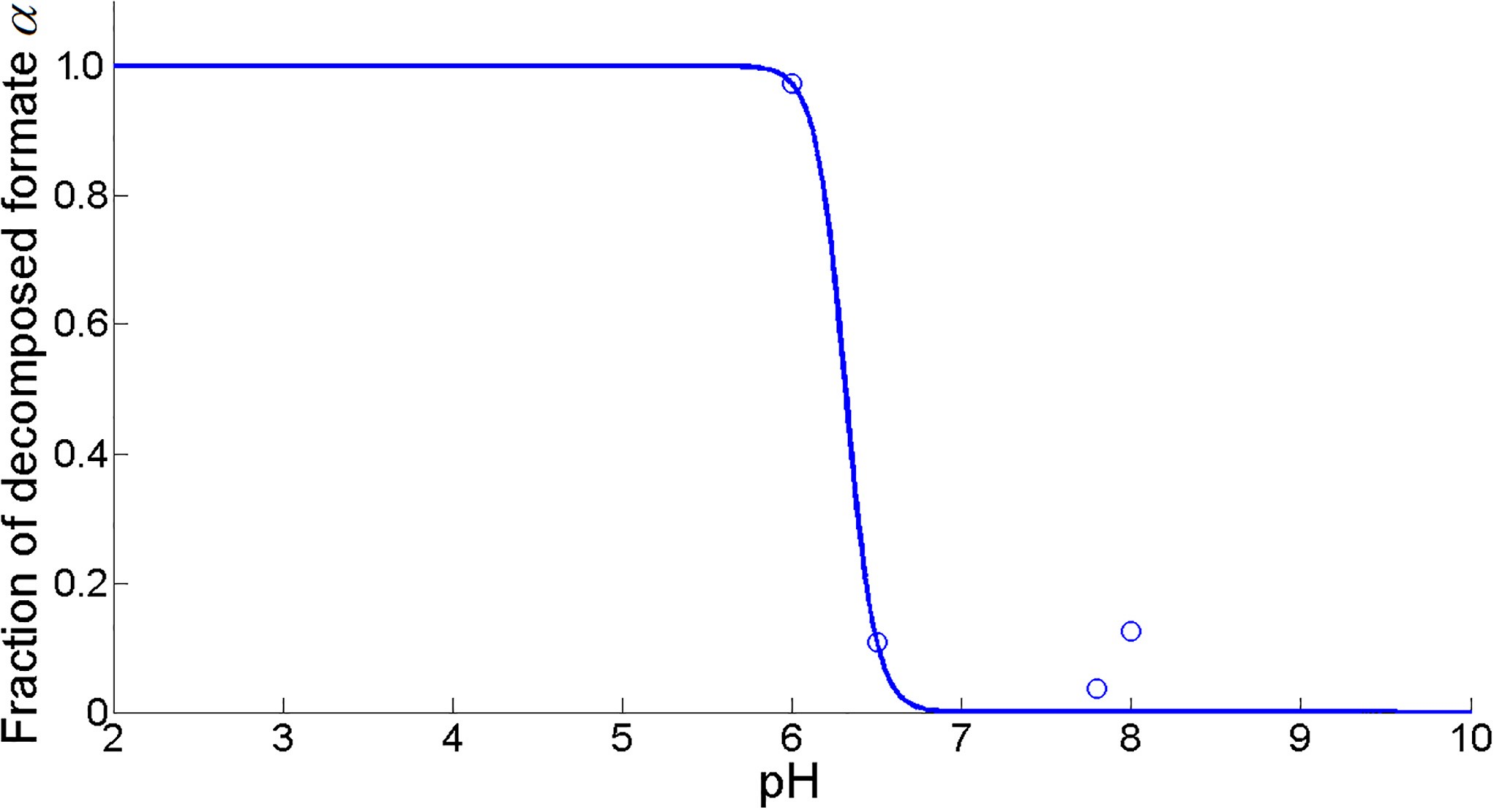
Fraction of decomposed formic acid *α* fit as a function of pH (MSSE = 0.0085). Experimental data (∘) are taken from [53], [59], and [61].

However, at low environmental pH values, formic acid from the PFL pathway is decomposed to carbon dioxide and dihydrogen by the formate hydrogen lyase (FHL) complex to limit internal acidification of the cell [51, 53]. It is assumed that the fraction of decomposed formic acid *α* follows a sigmoid curve as a function of pH (see Fig 7):

$$\begin{array}{c} \alpha \left(\text{pH}\right)=\frac{1}{1+\exp(11.3021\cdot (\text{pH}-6.3133))}. \end{array}$$ (26)

**Fig 8:**
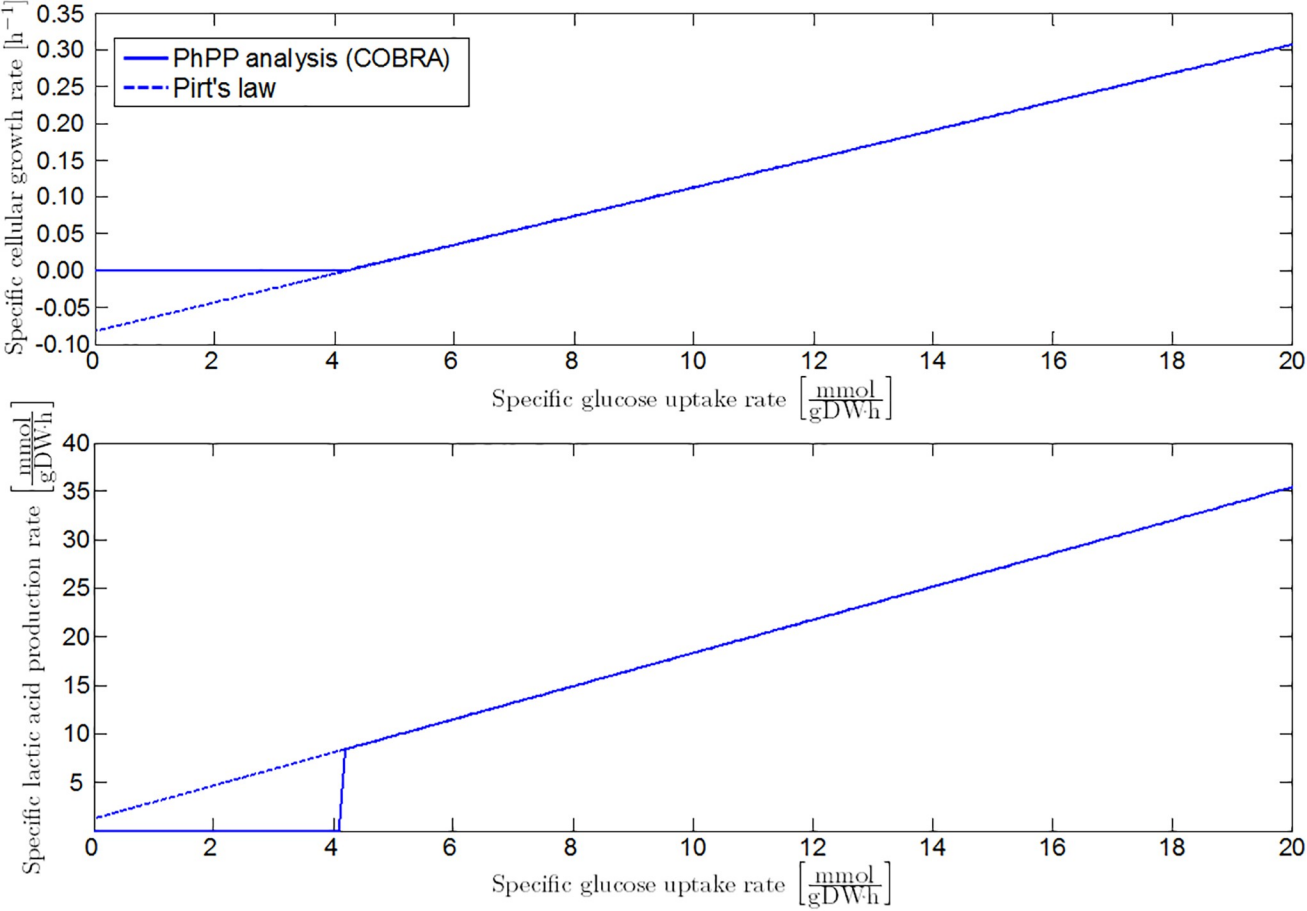
Specific growth rate and lactic acid secretion rate at the homolactic metabolic regime. Full lines are obtained from FBA with the iAF1260 model in which the PFL reaction is eliminated. The FBA results are fitted by the linear model of [23], as represented by the dashed lines.

In addition to formic acid decomposition, low environmental pH values result in the production of d-lactate by lactate dehydrogenase (LDH) [51, 54–57]. For every mole of acetic acid and two moles of formic acid generated under neutral conditions through the PFL pathway, two moles of less growth-inhibiting lactic acid are formed by the LDH enzyme. However, per mole of consumed glucose, the LDH metabolic pathway produces one mole of ATP less, due to the elimination of the acetate kinase (ACKA) reaction to acetic acid. Hence, the biomass yield coefficient at lactic acid production conditions $Y_{X/G}^{LDH}$ declines, while the reference maintenance coefficient $m_{G,ref}^{LDH}$ is higher compared to the PFL metabolism. Lactic acid production is simulated with the COBRA toolbox by eliminating the PFL (and aerobic PDH) reaction from the iAF1260 model (Fig 8).

**Fig 9:**
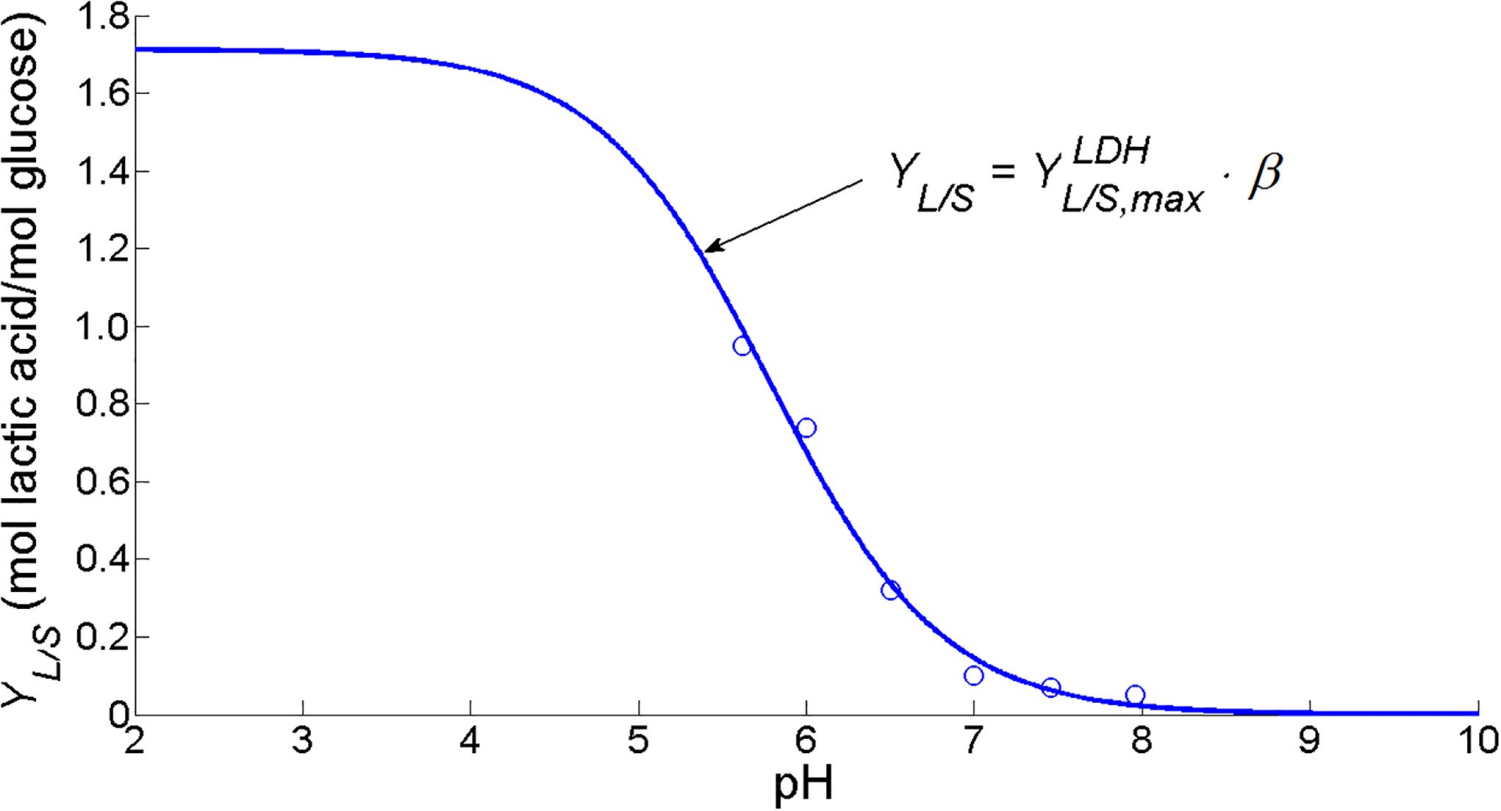
Lactic acid yield coefficient *Y*_*L*/*S*_ fitted as a sigmoid function of pH (MSSE = 0.0022). Experimental data (∘) are taken from [58]. The maximum lactic acid yield $Y_{L/S,max}^{LDH}$ in the homolactic LDH metabolic regime at low pH values is determined by means of an FBA analysis with the iAF1260 metabolic model [32] in the COBRA toolbox for MATLAB [33].

According to the iAF1260 model, the maximum yield of lactic acid on glucose is 1.713 moles of lactic acid per mole glucose when all glycolytic pyruvate is conducted through the LDH pathway. From data of [58], the dependence of lactic acid production on extracellular pH conditions is resolved. It is assumed that the lactic acid yield coefficient exhibits a sigmoid behavior as a function of extracellular pH, like the fraction of decomposed formic acid in the PFL pathway (Fig 9):

$$\begin{array}{c} Y_{L/G}=Y_{L/G}^{LDH}\cdot \beta \left(\text{pH}\right)=1.713\cdot \frac{1}{1+\exp(1.9547\cdot (\text{pH}-5.7809))}. \end{array}$$ (27)

Finally, the production of succinic acid from polyenolpyruvate (PEP) in Fig 6 is not taken into account. Secretion of succinic acid requires energy [27]. Hence, the production of succinic acid is negligible with respect to the other acid metabolites for a wild type *E. coli* strain [51, 53, 58, 59]. For a wild type *E. coli* strain, the yield of succinic acid on glucose is typically not higher than 0.2 moles of succinic acid per mole of consumed glucose [60].

#### Influence of extracellular pH and undissociated acid cell products on maintenance requirements

The dependence of the anaerobic maintenance coefficient on the extracellular proton and undissociated acid concentrations is composed of three terms, similarly to the aerobic case study (Eq (11)):

$$\begin{array}{c} m_{G}^{*}=m_{G,ref}^{*}+A^{*}\cdot \frac{\left[H^{+}\right]-10^{-7}}{{\left[H^{+}\right]}_{min}-10^{-7}}+B^{*}\cdot \sum_{i}\frac{\left[U_{i}\right]}{{\left[U_{i}\right]}_{min}^{an}}, \end{array}$$ (28)

where the * superscript denotes the metabolic regime of the microbial cells (LDH or PFL). For *E. coli*, the inhibitory proton concentration [H^+^]_*min*_ has the same value under aerobic and anaerobic conditions [38]. In contrast, MICs of undissociated acids under anaerobic conditions ${\left[{\text{U}}_{i}\right]}_{min}^{an}$ are in general slightly lower than their aerobic counterpart (see Table 1).

The A* and B* coefficients are defined in the same way as their aerobic equivalents (Eqs (12) and (14)):

$$A^{*}=q_{G,max}^{an}-m_{G,ref}^{*},$$ (29)

$$B^{*}=(q_{G,max}^{an}+\Delta q_{G/i}\cdot {\left[U_{i}\right]}_{min}^{an}-m_{G,ref}^{*}-A^{*}\cdot \frac{10^{-4.2}+10^{-5.4}-2\cdot 10^{-7}}{2\cdot ({\left[H^{+}\right]}_{min}-10^{-7})}).$$ (30)

under the assumptions that the cellular metabolism is homolactic (i.e., all glycolytic pyruvate is converted to lactic acid) for the calculation of *A*^*LDH*^ and *B*^*LDH*^, or that glycolytic pyruvate is completely converted through the PFL pathway for *A*^*PFL*^ and *B*^*PFL*^.

Values for the MIC of acetic, formic and lactic acid under anaerobic conditions are included in Table 1. Finally, the inhibitory effect of anaerobic ethanol production on microbial growth is not taken into account, as it is much lower than the growth-limiting influence of the lipophilic acids [62].

#### Combination of PFL and LDH metabolism

The sigmoid function *β* of Eq (27) is used to calculate a general biomass yield and maintenance coefficient for the combined PFL and LDH metabolism as a function of the extracellular pH:

$$\begin{array}{ccc} Y_{X/G} & = & \beta \cdot Y_{X/G}^{LDH}+\left(1-\beta \right)\cdot Y_{X/G}^{PFL}, \end{array}$$ (31)

$$\begin{array}{ccc} m_{G} & = & \beta \cdot m_{G}^{LDH}+\left(1-\beta \right)\cdot m_{G}^{PFL}, \end{array}$$ (32)

with $Y_{X/G}^{LDH}$ and $m_{G}^{LDH}$ the biomass yield coefficient and maintenance coefficient if the metabolic regime would be completely homolactic. The yield coefficient $Y_{X/G}^{PFL}$ and maintenance coefficient $m_{G}^{PFL}$ are representative for an exclusively PFL-mediated conversion of pyruvate.

Under anaerobic conditions, the additional required ATP production due to low pH values and the presence of undissociated acids is generated from the glycolytic EMP pathway and the secretion of mixed acid fermentation products. Hence, the total amount of acetic, formic, and lactic acid produced is calculated as follows:

$$q_{A}=\left(1-\beta \right)\cdot (Y_{A/G}^{PFL}\cdot q_{G}+\frac{m_{G}^{PFL}}{m_{G,ref}^{PFL}}\cdot q_{A,ref}^{PFL}{|}_{q_{G}=0}),$$ (33)

$$q_{F}=\left(1-\beta \right)\cdot (\left(1-\alpha \right)\cdot (Y_{F/G}^{PFL}\cdot q_{G}+\frac{m_{G}^{PFL}}{m_{G,ref}^{PFL}}\cdot q_{F,ref}^{PFL}{|}_{q_{G}=0})),$$ (34)

$$q_{L}=\beta \cdot (Y_{L/G}^{LDH}\cdot q_{G}+\frac{m_{G}^{LDH}}{m_{G,ref}^{LDH}}\cdot q_{L,ref}^{LDH}{|}_{q_{G}=0}).$$ (35)

### Case study III: Respiro-fermentative metabolism

#### Respiro-fermentative growth on glucose at reference conditions

**Fig 10:**
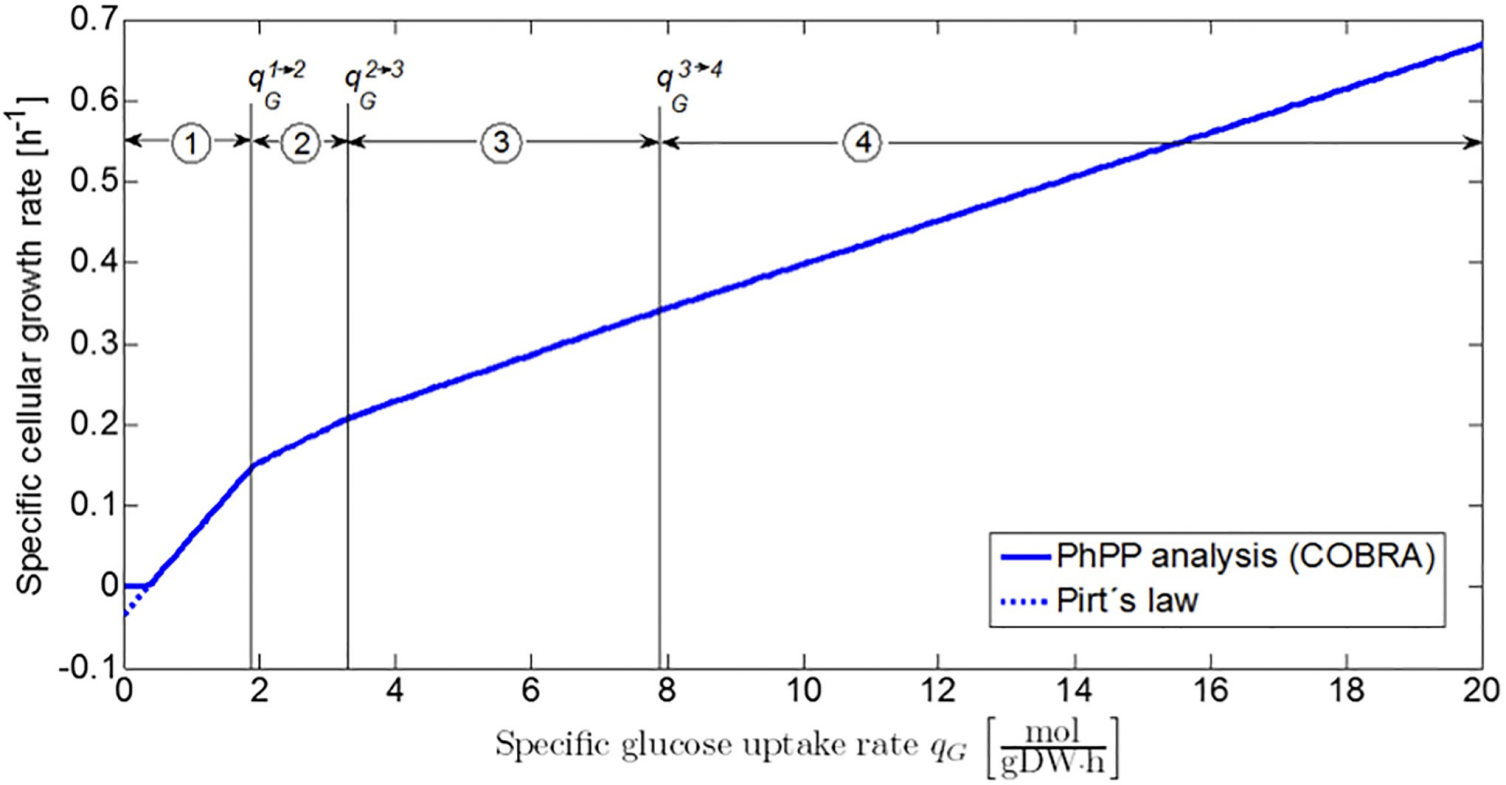
Specific growth rate as a function of specific glucose uptake rate at a constant specific oxygen uptake rate of 5 mmol/(gDW.h). The function exhibits a piecewise linear behavior in which each of the linear phases corresponds to one of the metabolic regimes (Sectors 1,2,3 and 4 in the PhPP in Fig 2).

In the previous two case studies, metabolic regimes at the extreme edges of the phenotypic phase plane are considered, viz. the respiratory metabolism at oxygen uptake rates higher than the line of optimality in Fig 2 (Sector 1) and the fermentative metabolism at anaerobic conditions. In between these two metabolic behaviors, the PhPP contains several respiro-fermentative metabolic regimes in which a range of weak acid products are produced by the microbial cells (Sectors 2, 3, and 4 in Fig 2). At a constant oxygen uptake rate, the specific growth rate exhibits a piecewise linear behavior as a function of the specific glucose uptake rate, as shown in Fig 10. Each of the linear phases corresponds to a metabolic regime characterized by its own biomass yield coefficient $Y_{X/G}^{\left(i\right)}$. Mathematically, the specific growth rate at a certain specific oxygen uptake rate is expressed as follows:

$$\begin{array}{ccc} \mu_{ref}^{\left(i\right)} & = & (q_{G}^{\left(1)\to (2\right)}-m_{G,ref}^{\left(i\right)})\cdot Y_{X/G}^{\left(1\right)}+\sum_{i=2}^{n-1}(q_{G}^{\left(i)\to (i+1\right)}-q_{G}^{\left(i-1)\to (i\right)})\cdot Y_{X/G}^{\left(i\right)}\\ & & +(q_{G}-q_{G}^{\left(n-1)\to (n\right)})\cdot Y_{X/G}^{\left(n\right)} \end{array}$$ (36)

in which *n* is the number of the PhPP sector correesponding to the (*q*_*G*_, *q*_*O*_) coordinates. The specific glucose uptake rates $q_{G}^{\left(i)\to (i+1\right)}$ corresponding to the boundary lines between two PhPP sectors or metabolic regimes *i* and *i* + 1 (e.g., $q_{G}^{\left(1)\to (2\right)}$ corresponds to the specific glucose uptake rate on the line of optimality).

#### Influence of extracellular pH and undissociated acid cell products on maintenance requirements

**Fig 11:**
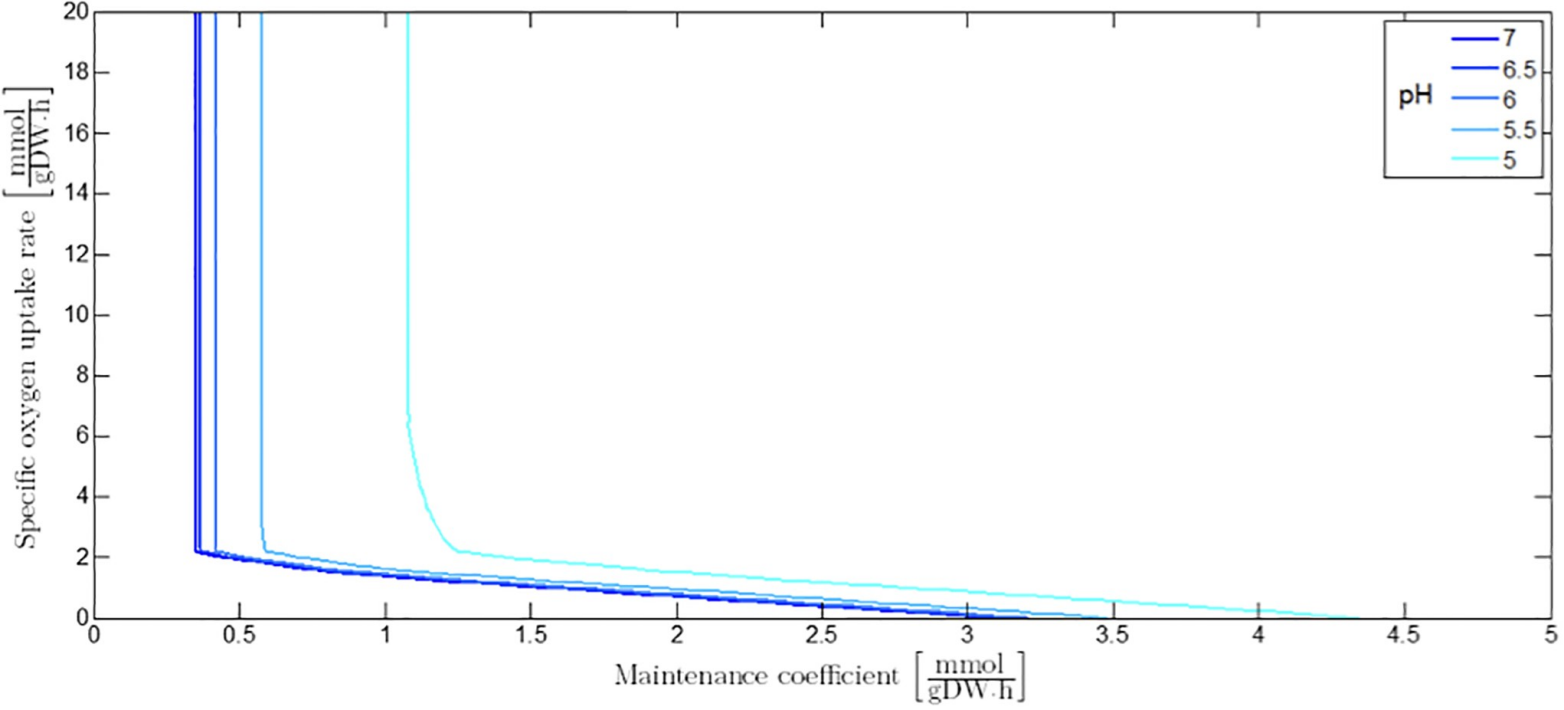
The influence of extracellular pH on the maintenance coefficient as a function of the specific oxygen uptake rate.

The influence of pH and undissociated acid cell products in the cellular environment can be taken into account by means of adaptation of the maintenance coefficient, analogously to Eqs (11) or (28). Decreasing the value of the extracellular pH results in the increase of the maintenance coefficient leading to a shift of the different sectors in the PhPP. However, the biomass yield coefficient in a specific PhPP sector stays constant as each of the PhPP sectors is associated to a specific metabolic regime. The maintenance coefficient at a certain value of the extracellular pH is illustrated as a function of the specific oxygen uptake rate in Fig 11. As can be derived from Eqs (11) or (28), similar graphs can be generated for the influence of undissociated acid cell products on the maintenance coefficient at a certain specific oxygen uptake rate (not shown here).

## Discussion

### Performance of the low-complexity model in comparison to FBA with the COBRA toolbox

To compare the run time of simulations with the developed low-complexity correlations versus explicit FBA computations, the case study of a fully aerobic batch experiment has been investigated. This batch experiment is mathematically described by the following equations:

$$\begin{array}{ccc} \frac{dC_{X}}{dt} & = & \mu \cdot C_{X}=\left(q_{G}-m_{G}\right)\cdot Y_{X/G}\cdot C_{X}, \end{array}$$ (37)

$$\begin{array}{ccc} \frac{dC_{G}}{dt} & = & -q_{G}\cdot C_{X}, \end{array}$$ (38)

with *C*_*X*_ [gDW/L] and *C*_*G*_ [mol/L] the concentrations of biomass and available glucose, respectively. An initial biomass and glucose concentration of respectively 0.1 gDW/L and 200 g/L are selected. The differential equations are solved by means of the ode45 solver of MATLAB^®^ [63]. For this solution, the time dimension is discretized in 100 equal intervals.

**Fig 12:**
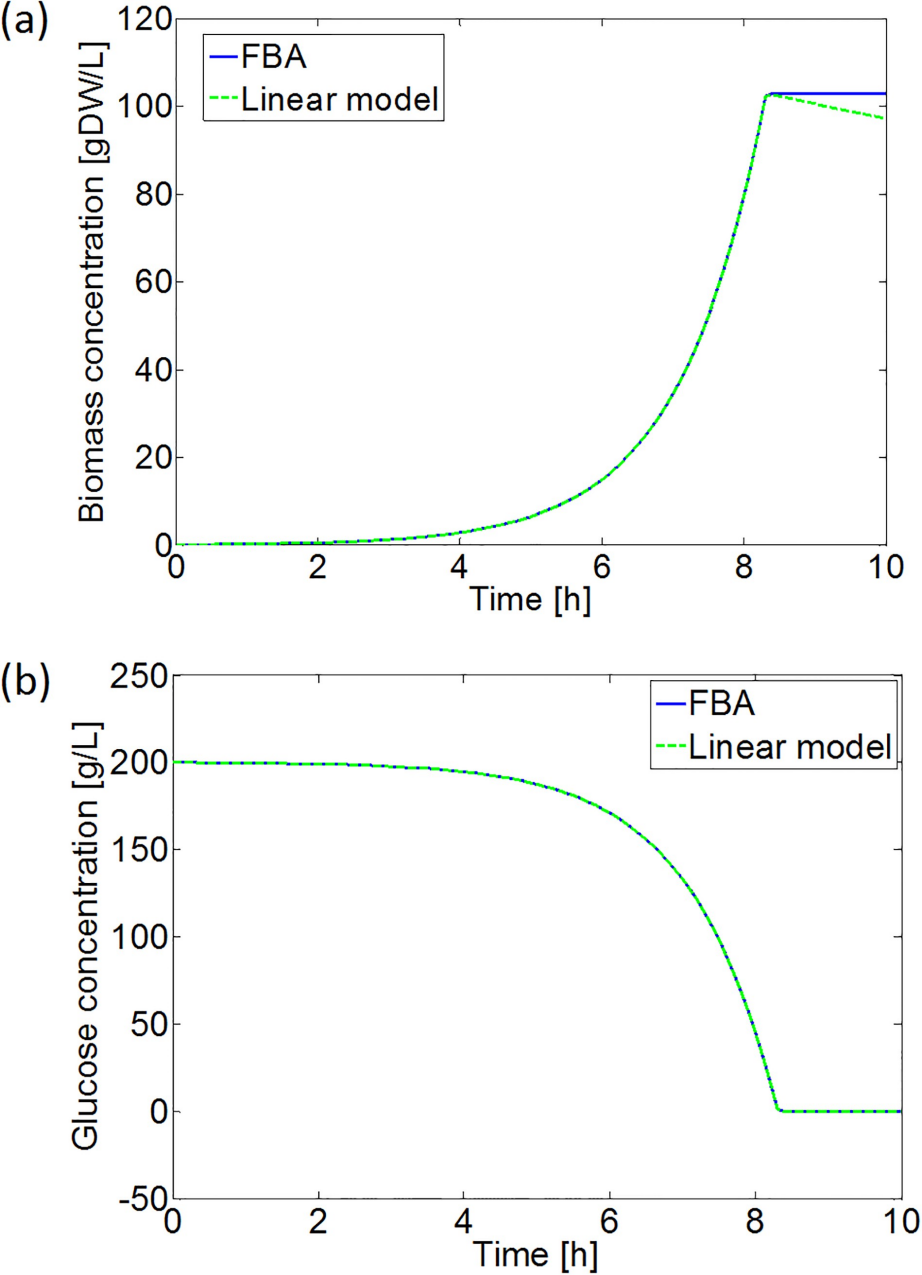
Simulation results of the aerobic batch experiment in Eqs (37) and (38) with FBA and the developed linear model: (a) biomass growth, and (b) glucose consumption. In Subfigure (a) the simulation with the FBA model does not exhibit declines in the biomass concentration, as the FBA with the COBRA toolbox is not capable to predict negative specific growth rates.

As expected, applying the linear correlation between the specific glucose uptake and growth rate reduces the required run time significantly. Without taking the initialization of the COBRA toolbox and the loading of the iAF1260 model into account, application of the linear correlation already results in a run time reduction of more than 99%. In addition, the simulation results are the same for both approaches, except when the specific glucose uptake rate drops below the critical value necessary to fulfill the cellular maintenance requirements. In the latter case, a zero specific growth rate is obtained from FBA, while the linear model predicts a negative growth (see Fig 12). However, the zero growth rate obtained from FBA is an artifact, merely indicating that no metabolic flux distribution resulting in a positive growth rate could be found.

As a conclusion, the developed linear model can be applied to provide fast and accurate predictive assessments of microbial bioprocesses, opening the way for real-time monitoring and robust control. Within this respect, the similarity between this model and the indirect coupling method of [64] should be stressed. In the method of [64], a look-up table is generated containing the FBA simulations constituting the reference PhPP in Fig 2. The specific cellular growth rate at a certain combination of specific glucose and oxygen uptake rates is determined by means of linear interpolation in this look-up table. However, the use of numerical data about the reference PhPP in table format renders it difficult to incorporate the influence of extracellular pH and undissociated acid cell products on the cellular growth. The advantage of our approach is that it is based on the use of linear equations with a biologically interpretable parameters (e.g., the maintenance coefficient) whose dependence on extracellular conditions can be easily understood and integrated in the model.

In addition, the linear model is deployable as an efficient tool for spatially-explicit individual-based simulations with a large number of microbial cells (e.g., to simulate microbial colony dynamics on/in semi-solid food products), in which knowledge about both cellular growth and local nutrient uptake from the cellular environment is necessary. This has been shown by the incorporation of the linear metabolic model in MICRODIMS, an in-house developed IbM platform for the simulation of microbial colony and biofilm dynamics, which has been described in [34].

### Novel approach to model the synergistic influence of stressing environmental conditions on microbial growth

The combined influence of environmental factors on microbial growth is traditionally modelled on the basis of the *gamma hypothesis* of [65]. This hypothesis states that different growth-inhibiting factors act independently. Mathematically, this implies that the effects of different environmental factors on microbial growth are multiplicative:

$$\begin{array}{c} \mu_{max}\left(pH,T,\left[I_{1}\right],...,\left[I_{n}\right]\right)=\mu_{opt}\cdot \gamma_{1}\left(pH\right)\cdot \gamma_{2}\left(T\right)\cdot \prod_{i=1}^{n}\gamma_{i+2}\left(\left[I_{i}\right]\right). \end{array}$$ (39)

In this formula, [I_*i*_] are the concentrations of growth-inhibiting substances, *μ*_*max*_ is the maximum specific growth rate under nutrient-rich conditions, and *μ*_*opt*_ is the maximum specific growth rate under optimal pH, temperature and weak acid concentrations. The gamma hypothesis has been used in, e.g., the model of [66], taking into account the separate influences of the environmental pH and the presence of undissociated and dissociated lactic acid on the maximum specific growth rate:

$$\mu_{max}\left(pH,\left[U_{L}\right],\left[D_{L}\right]\right)=\mu_{opt}\cdot (1-\frac{10^{pH_{min}}}{10^{pH}})\cdot (1-\frac{\left[U_{L}\right]}{{\left[U_{L}\right]}_{min}})\cdot (1-\frac{\left[D_{L}\right]}{{\left[D_{L}\right]}_{min}})$$ (40)

with *pH*_*min*_ the minimal pH value supporting microbial growth, [U_*L*_] and [D_*L*_] the respective concentration of undissociated and dissociated lactic acid in the system, and [U_*L*,*min*_] and [D_*L*,*min*_] the minimal concentrations of both lactic acid forms inhibiting growth. From the mathematical structure of Eq (40), it can be observed that there are no interactions between the different environmental factors.

However, the general applicability of the gamma hypothesis has been contested by experimental observations demonstrating that the growth-inhibiting effects of low pH values, undissociated acid concentrations, and high temperature are acting synergistically on microbial growth [35, 67–69], challenging the gamma hypothesis. Synergism between the growth-inhibiting conditions implies that, in contrast to the gamma hypothesis, the interval wherein a specific environmental factor allows microbial growth is dependent on the other factors. An accurate description of the interactions between different growth-inhibiting factors is important in terms of the hurdle technology, wherein several microbial stress factors are combined to find an optimal trade-off between organoleptic and nutritional quality, microbiological safety, and economic viability of food products [70].

To include the synergistic interaction between environmental stress factors, [67] adjusted Eq (39) with an interaction factor *ξ*:

$$\mu_{max}\left(pH,T,\left[I_{1}\right],...,\left[I_{n}\right]\right)=\mu_{opt}\cdot \gamma_{1}\left(pH\right)\cdot \gamma_{2}\left(T\right)\cdot (\prod_{i=1}^{n}\gamma_{i+2}\left(\left[I_{i}\right]\right))\cdot \xi \left(pH,T,\left[I_{1}\right],...,\left[I_{n}\right]\right).$$ (41)

**Fig 13:**
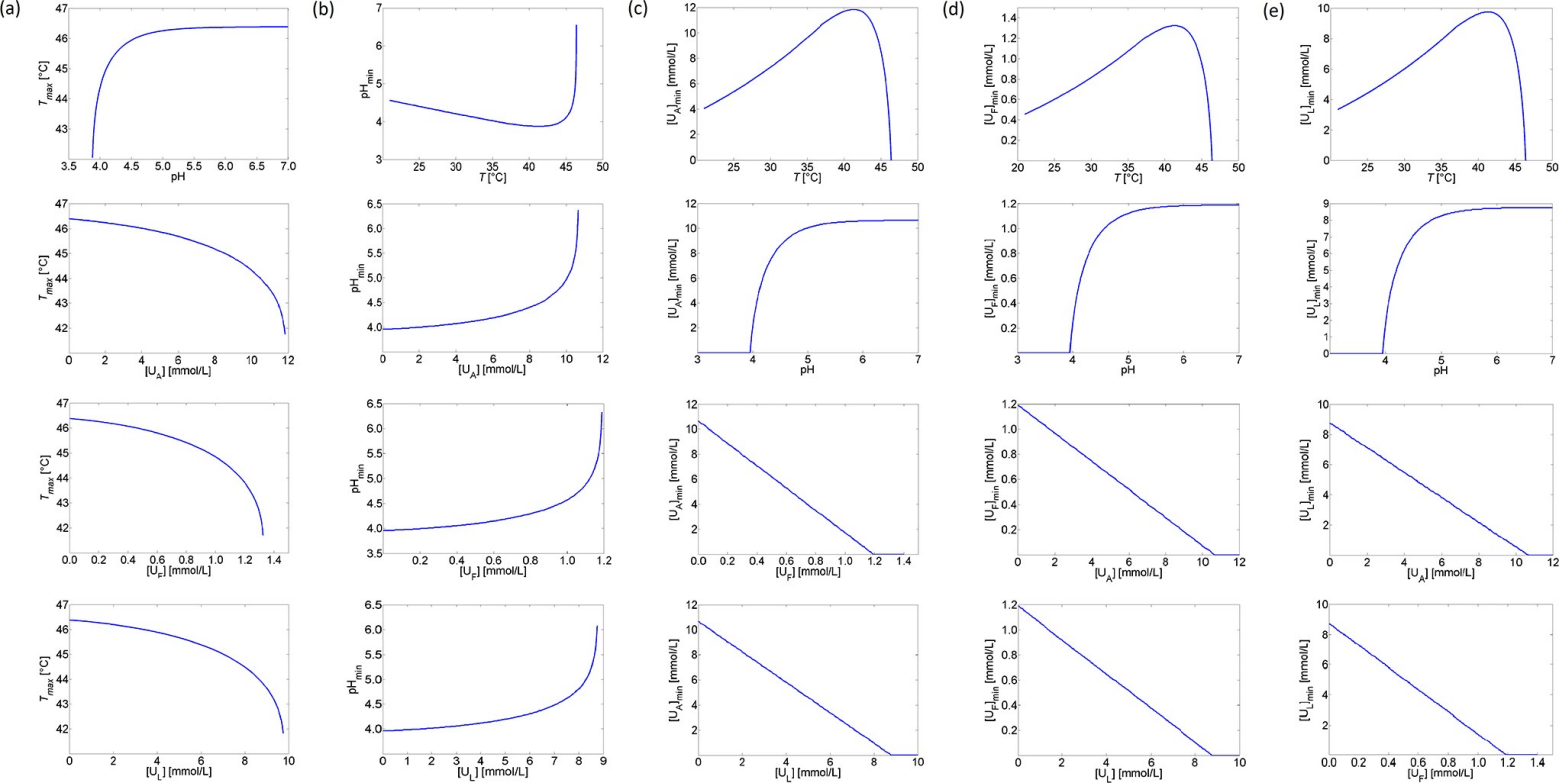
Interaction between cardinal parameters. (a) Maximum growth temperature *T*_*max*_ as a function of pH and undissociated acid concentrations; (b) *pH*_*min*_ as a function of temperature and undissociated acid concentrations; (c) Minimum inhibitory concentration of acetate [U_*A*_]_*min*_ as a function of temperature, pH, and formate and lactate concentrations; (d) Minimum inhibitory concentration of formate [U_*F*_]_*min*_ as a function of temperature, pH, and acetate and lactate concentrations; (e) Minimum inhibitory concentration of lactate [U_*L*_]_*min*_ as a function of temperature, pH, and formate and lactate concentrations.

In the current paper, a novel approach has been developed to incorporate the synergistic effects of different stress factors. Instead of extending the expression for the specific growth rate with an interaction factor, adjustment terms and factors are applied to the maintenance coefficient and the specific glucose uptake rate of *E. coli* to incorporate the influence of low pH values, undissociated organic acids, and high temperatures. Synergetic interactions between these environmental stress conditions are simulated for fully aerobic conditions and presented in Fig 13. The obtained concave down increase of *T*_*max*_ as a function of pH in the upper graph of Fig 13(a) is consistent with the experimental results of [35], while the increase of the undissociated acid MICs with pH (see the second graphs in Fig 13(c)–13(e)) matches with the observations of [38]. In Fig 13, it is demonstrated that the extreme value of a specific environmental condition (i.e., *T*_*max*_, *pH*_*min*_ or [U_*i*_]_*min*_) is less critical when the other environmental conditions are more optimal for growth.

The main advantage of the developed approach is that the synergistic effect of environmental factors on the maintenance coefficient and specific glucose uptake rate is explicitly incorporated in the metabolic models of the case studies. This makes the developed metabolic models particularly suitable to incorporate in spatially-explicit individual-based models taking local cellular glucose uptake from the environment into account.

### Further extensions to the developed metabolic model

In this work, a metabolic model has been developed for E. coli. The model may probably also be used for other food pathogens which are similar to E. coli, such as the gram-negative rod-shaped *Salmonella* Typhimurium for which a genome-scale model is available from [71], or even for metabolically engineered microorganisms producing valuable metabolic products, such as biocatalysts and bioactive molecules ([72, 73]).

In addition, to model microbial dynamics under glucose-limited conditions, the uptake of acetic and lactic acid as carbon source has to be taken into account. However, when the presence of organic acids in the food medium is only the result of glucose fermentation (i.e., weak acids are not initially present due to external addition), the concentrations of produced acids are too low to cause significant additional biomass growth.

Finally, the developed model is only valid for acid pH values and in a temperature range above 21°C. To model microbial growth under 21°C, an additional function has to be added to the piecewise expression in Eqs (20) and (21). This additional function should be arranged such that (i) at 21°C, there is a smooth transition between this function and the function in Eq (20), and (ii) at lower temperatures of around 0-5°C, the specific microbial growth rate goes to zero.

### Conclusions

In predictive microbiology, accurate descriptions of microbial growth dynamics are the primary goal. However, some other, more practical concerns need to be taken into account, like the required simulation time, model complexity and genericness. Fast simulation run times are indispensable for real-time monitoring and contol of microbial bioprocesses, implying that the cellular metabolism needs to be modeled in the least complex way that is still accurate enough for the considered biosystem, as complex metabolic models lead to high computational loads and simulation run times. In the same way, the least complex metabolic model needs to be applied to individual-based simulations of multiple cell systems, as each cell is simulated separately. Furthermore, when the individual-based simulation is spatially-explicit, attention should be paid to the genericness of the metabolic model in order to simulate the impact of heterogeneous and dynamic environmental conditions on the cellular growth dynamics accurately.

In this paper, metabolic models are built for the description of *E. coli* growth dynamics under aerobic, micro-aerobic, and anaerobic conditions, based on the linear dependency between the specific microbial growth and nutrient consumption rate of [23]. Although their low-complexity structure, these models can be completed without empirical calibration with information about the intracellular cell metabolism by fitting them to the results of flux balance analyses (FBA) with the genome-scale iAF1260 model. In this way, the intended trade-off between model complexity, accuracy and genericness is achieved.

Flux balance analyses with iAF1260 are only valid for specific reference environmental conditions, viz., a neutral M9 medium enriched with glucose at a temperature of 37 °C. The influences of low pH values, weak acid cell products and high temperatures as growth-inhibiting environmental factors have been incorporated in the form of adjustment terms or factors on the maintenance coefficient or specific nutrient uptake rate in Pirt’s law. For anaerobic conditions, the shift to a lactic acid producing metabolism at low pH values has been implemented as well. This leads to a novel and more intuitive approach to simulate the synergistic effect of the considered microbial stress conditions on microbial growth. As a result, this approach is an excellent low-complexity tool within the context of hurdle technology to find combinations of growth-inhibiting conditions optimizing quality, safety and, economic value of food products.

## Supporting information

S1 Text (PDF)
