# Supplementary material for: A low-complexity metabolic network model for the respiratory and fermentative metabolism of Escherichia coli
Source: PLoS One. 2018 Aug 29;13(8):e0202565. doi: 10.1371/journal.pone.0202565 (PMC6114798; doi:10.1371/journal.pone.0202565)
Supplement: S1 Text — (PDF) [file pone.0202565.s001.pdf]

S1 Text

In this appendix, all mathematical equations developed in this paper are summarized in a schematic and accessible way. Under both aerobic and anaerobic conditions the following differential equations hold for the substances present in the medium:

$$\begin{aligned}
\text{Biomass growth:} \quad & \frac{\mathrm{d}C_X}{\mathrm{d}t} = \mu \cdot C_X = (q_G - m_G) \cdot Y_{X/G} \cdot C_X \\
& = (q_{G,max} \cdot \frac{C_G}{K_G + C_G} - m_G) \cdot Y_{X/G} \cdot C_X \\
\text{Glucose consumption:} \quad & \frac{\mathrm{d}C_G}{\mathrm{d}t} = -q_G \cdot C_X \\
\text{Acetic acid excretion:} \quad & \frac{\mathrm{d}C_A}{\mathrm{d}t} = q_A \cdot C_X \\
\text{Formic acid excretion:} \quad & \frac{\mathrm{d}C_F}{\mathrm{d}t} = q_F \cdot C_X \\
\text{Lactic acid excretion:} \quad & \frac{\mathrm{d}C_L}{\mathrm{d}t} = q_L \cdot C_X
\end{aligned}$$

Under fully aerobic conditions (Sector 1 in Fig. ??), the maintenance coefficient m_G consists of three terms:

$$m_G^{(1)} = m_{G,ref}^{(1)} + A^{(1)} \cdot \frac{[\mathrm{H}^+] - 10^{-7}}{[\mathrm{H}^+]_{min} - 10^{-7}} + B^{(1)} \cdot \sum_i \frac{[\mathrm{U}_i]}{[\mathrm{U}_i^a]_{min}},$$

with $[\mathrm{H}^+]$ and $[\mathrm{U}_i]$ respectively the concentration of protons and undissociated acids in the medium. The constants $A^{(1)}$ and $B^{(1)}$ are calculated as follows:

$$\begin{aligned}
A^{(1)} &= q_{G,max}^a - m_{G,ref}^{(1)}, \\
B_A^{(1)} &= q_{G,max}^a + \Delta q_{G/A} \cdot [\mathrm{U}_A]_{min}^a - m_{G,ref}^{(1)} \\
&\quad -A^{(1)} \cdot \frac{10^{-4.2} + 10^{-5.4} - 2 \cdot 10^{-7}}{2 \cdot ([\mathrm{H}^+]_{min} - 10^{-7})}.
\end{aligned}$$

Under fully aerobic conditions, *Escherichia coli* cells do not excrete any weak acid into their surroundings, meaning that

$$q_A = q_F = q_L = 0 \ \tfrac{\mathrm{mol}}{\mathrm{gDW \cdot h}}.$$

Under anaerobic conditions, the maintenance and biomass yield coefficient are determined by a linear combination of LDH and PFL metabolic pathways (see Fig. ??):

$$\begin{aligned}
Y_{X/G} &= \beta \cdot Y_{X/G}^{LDH} + (1 - \beta) \cdot Y_{X/G}^{PFL}, \\
m_G &= \beta \cdot m_G^{LDH} + (1 - \beta) \cdot m_G^{PFL}.
\end{aligned}$$

Maintenance coefficients for the LDH and PFL metabolism are calculated in similar way to the maintenance coefficient under fully aerobic conditions. The variable β represents the fraction of pyruvate converted to lactic acid, and is a function of the extracellular pH:

$$\beta(\mathrm{pH}) = \frac{1}{1 + \exp(1.9547 \cdot (\mathrm{pH} - 5.7809))}.$$

The specific excretion rates of acetic, formic, and lactic acid under anaerobic conditions is expressed as follows:

$$\begin{aligned} q_A &= (1-\beta)\cdot\left(Y_{A/G,max}\cdot q_G+\frac{m_G^{PFL}}{m_{G,ref}^{PFL}}\cdot q_{A,ref}^{PFL}|_{q_G=0}\right), \\ q_F &= (1-\beta)\cdot\left((1-\alpha)\cdot\left(Y_{F/G,max}\cdot q_G+\frac{m_G^{PFL}}{m_{G,ref}^{PFL}}\cdot q_{F,ref}^{PFL}|_{q_G=0}\right)\right), \\ q_L &= \beta\cdot\left(Y_{L/G,max}\cdot q_G+\frac{m_G^{LDH}}{m_{G,ref}^{LDH}}\cdot q_{L,ref}^{LDH}|_{q_G=0}\right), \end{aligned}$$

with α indicating the fraction of formic acid that is intracellularly converted to dihydrogen and carbon dioxide as a function of extracellular pH:

$$\alpha(\mathrm{pH})=\frac{1}{1+\exp(11.3021\cdot(\mathrm{pH}-6.3133))}.$$

Values for all parameters in the previous mathematical expressions are listed in Table **??**.
